# Large-Scale Carbon Removal Will Create Public Health, Economic, and Climate Trade-Offs

**DOI:** 10.1021/acs.est.5c17950

**Published:** 2026-06-08

**Authors:** Parisa Javadi, Patrick O’Rourke, Jay Fuhrman, Daniel H. Loughlin, Scott C. Doney, William Shobe, João Ferreira, Andrés F. Clarens

**Affiliations:** † Department of Civil and Environmental Engineering, 2358 University of Virginia, Charlottesville, Virginia 22904, United States; ‡ University of Maryland, College Park, Maryland 20742, United States; § Center for Global Sustainability, 205814 University of Maryland, College Park, Maryland 20742, United States; ∥ Nicholas School of the Environment, Duke University, Durham, North Carolina 27708, United States; ⊥ Department of Environmental Sciences, University of Virginia, Charlottesville, Virginia 22904, United States; # Batten School of Leadership and Public Policy, 2358 University of Virginia, Charlottesville, Virginia 22904, United States

**Keywords:** carbon dioxide removal, decarbonization, climate change mitigation, air pollution, public health, integrated assessment modeling

## Abstract

Economy-wide efforts to achieve net-zero emissions offer climate and air quality-related public health benefits from reducing fossil fuel combustion. However, carbon dioxide removal (CDR) may be necessary to meet emissions targets cost-effectively, and relying on CDR would forego some air-quality benefits. Here, we systematically quantify the regional air quality and public health implications of six CDR portfolios for the U.S. using a coupled modeling approach and compare those to a no U.S. climate action scenario. While both high- and low-CDR deployment avoid about $2.5–5.8 trillion USD2020 (or 0.4–0.8% of cumulative GDP (CGDP)) in climate damages, the high-CDR pathway costs $11–13 trillion USD2020 (or 1.8–1.9% of CGDP) by 2050, whereas the low-CDR pathway costs $16–20 trillion USD2020 (or 2.6–2.9% of CGDP) due to deeper near-term fossil fuel reductions. Public health benefits reach $2.8–6.5 trillion USD2020 (or 0.5–0.9% of CGDP) under high-CDR and are $3.5–8 trillion USD2020 (0.6–1.2% of CGDP) under low-CDR, reflecting greater reductions in particulate matter and ozone exposure and preventing approximately 12,600 additional premature deaths by mid-century. However, heavy reliance on CDR technologies could generate $5–6 trillion USD2020 (∼0.8–0.9% of CGDP) in CDR revenues by 2050, exemplifying the trade-offs between public health, economy, and climate.

## Graphical abstract

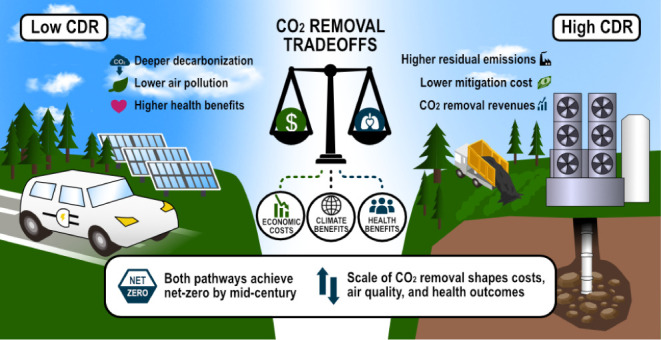

## 1 Introduction

Fossil fuel combustion is the principal source of both greenhouse gas (GHG) emissions and criteria air pollutants such as particulate matter (PM_2.5_) and ozone (O_3_).^1^ Efforts to reduce climate pollution are expected to have concomitant benefits of reducing air pollutant emissions and improving public health.^2–5^ In the U.S., air pollution causes 100,000–200,000 premature deaths annually, valued at $0.9–1.8 trillion USD2020 based on the Environmental Protection Agency (EPA) $9.25 million USD2020 value of statistical life.^6^ Recent estimates suggest that U.S. climate and clean-energy policies passed in 2022 and rescinded in 2025 would have averted between 3,400 and 20,000 of these premature deaths per year by 2030.^7–11^ While these estimates are sensitive to policy design, sociodemographic projections, and exposure–response functions, the connection between decarbonization and public health is robust.^12–17^

Strategies for mitigating GHG emissions are increasingly organized in terms of net-zero by mid-century plans.^18,19^ These plans often depend on atmospheric carbon dioxide removal (CDR) since hard-to-abate sectors are more expensive to decarbonize directly than to offset by CDR.^20^ While large-scale CDR may keep decarbonization costs manageable under a fixed economy-wide net emissions target, policy designs that allow CDR to substitute for gross emission reductions may slow fossil fuel phase-out and increase energy demand for CDR technologies.^21,22^ Part of this demand would likely be met with fossil fuels, creating further GHG and air pollutant emissions;^23–27^ but these effects would not necessarily arise under policy architectures that impose binding constraints on gross emissions and CDR. Given the links among carbon emissions, air pollution, and public health, we hypothesize that the scale of CDR deployment will influence the health outcomes of achieving net-zero emissions.^28–37^ Moreover, different CDR types have distinct pollutant profiles, and their scale, timing, and permanence will all shape these outcomes.^38–43^ High and low reliance on CDR represent distinct but plausible policy pathways. Large-scale, near-term deployment of CDR technologies provides a cheaper flexible decarbonization pathway that can potentially lead to net-negative emissions later in the century, particularly if CDR technologies scale rapidly and become cost-effective.^17,44,45^ In contrast, mitigation deterrence under a fixed net emission target, CDR sustainability risks, and emerging evidence of prudent limits to geological CO_2_ storage motivate futures with lower CDR deployment.^46–49^

Achieving U.S. carbon neutrality by 2050 is projected to require 0.8–2.9 GtCO_2_ yr^–1^ of gross removal.^38–41^ These estimates depend on uncertain techno-economic factors such as CDR costs, CO_2_ storage capacity, and land suitability for enhanced rock weathering (ERW).^42,43^ CDR can offset residual emissions from hard-to-abate sectors, offering one of the many mechanisms for shifting climate trajectories, particularly when deep reductions in gross CO_2_ and non-CO_2_ emissions become expensive.^50,51,52^ Most existing analyses consider only direct air capture and bioenergy with carbon capture and storage (DACCS and BECCS) options,^41,53^ limiting understanding of how a broader CDR portfolio could influence air pollution and public health outcomes. Recent work explores some of the anticipated trade-offs among CDR approaches (e.g., in technological costs, maturity, cobenefits, environmental and social factors).^17,54–58^

The large-scale deployment of CDR can have unintended effects on water, land, and energy systems. When BECCS is heavily relied on, regional water consumption can increase for biomass crops irrigation. Projections show that in the U.S. by the mid-century, some states will use more than 10% of their total water use for BECCS.^41^ In some southern states, land allocation for BECCS can potentially affect agricultural land allocation, as well as food prices and supply.^41,59^ Increased reliance on DACCS introduces additional pressure on energy systems, as this technology requires large amounts of electricity and/or natural gas, requiring the expansion of renewable and natural gas with CCS.^21,41,59,60^

Recent analysis has also showed that diversified CDR portfolios can balance land, energy, and economic burdens, reducing reliance on single options and lowering policy costs,^41,61^ and have highlighted that at the global scale minimizing CDR dependence remarkably reduces GHG emissions and air pollutants.^22^ The climate benefit of CDR comes with complex trade-offs that could undermine climate and health objectives if not carefully managed. However, existing literature rarely goes beyond air pollution and the physical implications of high vs low CDR approaches to evaluate at the national level how differing levels of CDR deployment reshape simultaneously the climate, public health, and economic outcomes of decarbonization. Reliance on different CDR methods and the resulting shifts in resource use will require varying infrastructure and investment levels, creating complex trade-offs. These differences shape how quickly and where CDR technologies are deployed, their interaction with decarbonization policies, and their abatement and cobenefit cost profiles. Understanding these dynamics demands an integrated modeling framework linking CDR techno-economics, sectoral emissions trajectories, resulting climate forcing, mitigation costs, and public health cobenefits from improved air quality.

To address these concerns about the trade-offs and implications of high vs low CDR futures, we developed such a framework that combines three models. First we utilize the Global Change Analysis Model (GCAM),^62^ an integrated multisector model developed and maintained at the Pacific Northwest National Laboratory’s Joint Global Change Research Institute (JGCRI). GCAM simulates detailed energy-water-land-climate-economy interactions, projecting technological deployment, energy supply and demand, sectoral and regional GHG and non-GHG emissions, and marginal abatement cost curves under alternative policy and techno-economic assumptions. GCAM scenarios are often used in Working Group III contributions to IPCC reports.^63^ In earlier work, we modeled a diverse set of CDR approaches in GCAM at both the global and U.S. state levels; the model used here includes DACCS, BECCS, LUC, DOCCS, ERW, and biochar as the CDR approaches (Table 1).^41,53,59,60^ This study uses a U.S.-focused version of GCAM called GCAM-USA, enabling us to offer more granular analysis of state-level emissions trajectories within the context of the larger multiregional global representation.

**Table 1:** Description of CDR Technologies Available in This study^41,53^

CDR approach	CDR technology	Description
Nature-based	Land-use Change (LUC)	Sequestration of atmospheric CO_2_ through afforestation and reforestation
Engineered	Direct Air Carbon Capture and Storage (DACCS)	Operation of solvent or sorbent-based facilities using combination of electricity and natural gas to separate and store atmospheric CO_2_ in geological reservoirs
Combined nature and engineered	Bioenergy with Carbon Capture and Storage (BECCS)	Production of biomass for industrial energy use and/or to generate electricity, liquid fuels, and hydrogen, while capturing and storing CO_2_ in geological reservoirs
	Soil Enhancement Using Biochar	Generation of biochar through the slow-pyrolysis process of second-generation biomass and application on croplands as part of the crop cultivation practices
	Enhanced Rock Weathering (ERW)	Application of crushed basalt on croplands to remove atmospheric CO_2_
	Direct Ocean Carbon Capture and Storage (DOCCS)	Electrochemical stripping of CO_2_, either stand-alone or colocated with desalination units, paired with geological CO_2_ storage

We then used the Finite-amplitude Impulse-Response (FaIR) model,^64^ a reduced-complexity climate model, to translate GHG and aerosol forcing pathways into probabilistic projections of global mean surface temperature change relative to preindustrial baselines. We also employed the recent U.S. EPA estimate of the damages from anthropogenic GHG emissions,^65^ which provides a standardized approach to monetize avoided climate damages from reduced GHG emissions. Finally, we used the EPA Co-Benefits Risk Assessment (COBRA)^66^ tool, which includes a reduced-complexity source–receptor air quality and health assessment model, to quantify changes in ambient PM_2.5_ and ground-level O_3_ concentrations and to estimate the associated premature mortality and monetized health benefits.^67^

We analyze three scenarios. The baseline scenario, No Climate Action in USA (NCA-USA), assumes that U.S. federal policy includes no economy-wide climate measures while other nations progress collectively toward a mid-century net-zero CO_2_ emission target. We contrasted this baseline against our High- and Low-CDR scenarios, both of which achieve U.S. and global net-zero CO_2_ emissions by 2050. This approach allowed us to investigate how different scales of CDR deployment could shape global radiative forcing and surface temperature rise relative to preindustrial levels, as well as to explore impacts on emissions trajectories, CO_2_ mitigation costs, CDR revenues, air quality improvements, and climate damages compared to the absence of coordinated U.S. national climate policy.

## 2 Methods

We use multiple data sets and a three-model framework to assess global and U.S. decarbonization pathways under different scales of CDR deployment. We employ the most recent EPA national level estimate of marginal climate damages from GHGs^65^ to monetize avoided climate damages. We use GCAM-USA,^62^ an integrated assessment model (IAM) that captures detailed energy–land–economy feedbacks, projects technological deployment, energy supply and demand, sectoral emissions, and marginal abatement cost curves under alternative policy and techno-economic assumptions. Our GCAM implementation explicitly represents a suite of six CDR options on both global and state scales. We compute CO_2_ mitigation costs, CDR revenues, and net mitigation costs under our decarbonization scenarios. We feed GCAM’s emissions trajectories into the FaIR^64^ model, a computationally efficient climate emulator that converts GHG and aerosol forcings into probabilistic projections of global mean surface temperature relative to preindustrial baselines. Finally, we apply the EPA’s COBRA^66^ model, a reduced-complexity source–receptor air quality and public health model, to estimate changes in ambient PM_2.5_ and O_3_, calculate associated premature mortality and morbidity, and monetize the resulting health benefits. Full methodological details are detailed in the subsequent subsections. Descriptions of CO_2_ mitigation costs as well as CDR costs and revenues analysis are provided in SI sections S.1.E and S.1.F.

### 2.1 GCAM Module

GCAM is an open source IAM developed and maintained by the Joint Global Change Research Institute (available at: https://github.com/JGCRI/gcam-core/releases). GCAM simulates the dynamics and interdependencies among energy, water, land, economy, and climate systems across 32 geopolitical regions worldwide and computes equilibrium prices and input-output quantities for all markets in each region and modeling period. GCAM-USA is an extension of the global model, providing finer resolution by disaggregating the U.S. into 50 states and the District of Columbia. We use the open-source release of GCAM-USA v7.1. Documentation is accessible via the model’s documentation page (https://jgcri.github.io/gcam-doc/gcam-usa.html). Description of energy systems and emissions projections in GCAM-USA are provided in SI section S.1.G.

#### 2.1.1 CDR Technologies Representation

Previously we enhanced GCAM at the global and U.S. states levels to include a diverse set of CDR approaches;^41,53,59,60^ therefore, in this study we analyze the decarbonization pathways harnessing the capabilities of DACCS, LUC, BECCS, ERW, biochar soil enhancement, and DOCCS.

For DACCS, which combines capture of atmospheric CO_2_ and subsurface storage, both solvent-based, high-temperature DACCS and solid-sorbent, low-temperature DACCS are represented.^68,69^ In GCAM, both types of DACCS become more efficient and less costly over time, building on prior DACCS implementations.^59,70^ Electricity demand for electricity-based DACCS is supplied by the grid and reflects the regional least-cost power generation mix, rather than plant level procurements (e.g., via power purchase agreements). As a result, electricity use for this technology affects the overall generation mix endogenously, with grid carbon intensity determined by the binding policy constraint rather than by contractual sourcing assumptions. LUC or afforestation and reforestation is modeled by converting available nonforested or recently deforested lands into forests, with regionally varying growth rates and sequestration potentials.^71^ BECCS couples biomass cultivation, which competes with food crops cultivation and often requires irrigation and fertilizers, with energy generation and CO_2_ capture. GCAM explicitly tracks the land area for biomass feedstocks, energy conversion efficiencies, and resulting CO_2_ sequestration, while assessing BECCS competitiveness, regional biomass availability, and storage access at the state level. The state-level CO_2_ abatement cost in 2050 for novel CDR technologies in GCAM-USA are provided in the SI (Figure S.1).

ERW involves the application of finely ground basalt on croplands, where accelerated silicate weathering sequesters atmospheric CO_2_ and generates dissolved inorganic carbon. These dissolution products are partitioned across soil storage, groundwater, and surface runoff, and over decades to centuries are exported to inland waters and, ultimately, the ocean.^72,73^ U.S. state-level ERW supply curves are downscaled from a national cost curve,^74^ with an added nonenergy cost component that covers capital, operation and maintenance, mining, grinding, transport, and spreading costs.^41,53^ Details of ERW model development in GCAM-USA are provided in Javadi (2024).^41^

Biochar, produced via slow pyrolysis of biomass, locks carbon into soils for centuries and can boost soil fertility and crop yields.^75,76^ GCAM-USA treats biochar application as a competing cropland practice, with per acre yields varying by climate zone, and uses parameters for production cost, carbon yield fraction, byproduct generation, and application rates.^77^ Feedstocks are limited to residues and marginal-land crops to avoid food-land competition.

In the case of DOCCS, electrochemical separation of seawater into acidic and alkaline streams elevates ocean CO_2_ uptake, with captured CO_2_ that gets stored geologically.^78,79^ We model both stand-alone DOCCS and units colocated with desalination which limits the removal to less than 100 ktCO_2_ yr^–1^ based on the size of the current largest desalination plant.^41,53,80^ These technologies remove CO_2_ directly from the atmosphere and therefore contribute to net negative emissions.

Detailed techno-economic assumptions for CDR technologies follows our previous work.^41^ We have conducted a detailed accounting on non-CO_2_ emissions for postcombustion CCS and the supply chain of the modeled CDR technologies, available in SI section S.1.C.

#### 2.1.2 Scenario Design in GCAM

We modeled a baseline scenario where the U.S. does not consider any climate policy or action (NCA-USA scenario), but the rest of the world reaches net-zero CO_2_ emissions in 2050. We also modeled two global and the U.S. net-zero emissions scenarios differentiated by their reliance on CDR: a High-CDR and a Low-CDR scenario. We implement a net-zero CO_2_ constraint by 2050 rather than a net-zero GHG target as a stylized deep mitigation scenario designed to isolate the role of CDR. LUC removals are not included in the economy-wide CO_2_ emissions constraint because of uncertainties regarding permanence of this CDR approach. Thus, in this analysis LUC provides additional removal. These scenarios varied the deployment scale of the six CDR technologies through adjustments to land carbon pricing, availability of CCS, biomass resources, and deployment of ERW, reflecting constrained versus unconstrained CDR deployments to achieve net-zero emissions by 2050. The scenarios are based on the assumptions of the Shared Socioeconomic Pathway 2, which includes medium population growth (∼9 billion in 2100), medium income and technological development, production and consumption patterns are a continuation of past trends, and only a gradual reduction in inequality occurs.^81^

Table 2 provides technical assumptions regarding the modeled scenarios. In the High-CDR scenarios, we applied a constraint on the future deployment of ERW which is 50% lower compared to the removal levels seen before the constraint was introduced due to potential impacts on local air quality during the application of rocks on croplands. Due to the food price implications of dedicating a large area of agricultural lands to bioenergy cultivation, we assumed 50% lower biomass availability than would have otherwise deployed without any constraints in 2050. Thus, we assumed biomass availability increases linearly from 31 EJ in 2025 to 62.5 EJ in 2050. We set a land carbon price that starts at zero in 2020 and linearly reaches half of the carbon price imposed on the energy system’s CO_2_ emissions by 2050.

**Table 2:** Modeling Assumptions for the Three Decarbonization Scenarios

	Scenario		
Constraint	NCA-USA	High-CDR	Low-CDR
Global CO_2_ policy	32 GtCO_2_ yr^–1^ in 2025 linearly to 0 in 2050		
U.S. national CO_2_ policy	No constraint	4.3 GtCO_2_ yr^–1^ in 2025 linearly to 0 in 2050	
Global biomass constraint		31 EJ yr^–1^ in 2025 linearly to 62.5 EJ yr^–1^ in 2050	Constant 31 EJ yr^–1^ through 2050
Global ERW constraint		0 in 2020 linearly to 1.62 GtCO_2_ yr^–1^ in 2050	0 in 2020 linearly to 324.13 MtCO_2_ yr^–1^ in 2050
National ERW constraint		0 in 2020 linearly to 337.07 MtCO_2_ yr^–1^ in 2050	0 in 2020 linearly to 64.15 MtCO_2_ yr^–1^ in 2050
Global CCS constraint		No constraint	3 GtCO_2_ yr^–1^ from 2030 to 2050
The fraction of the price on energy emissions applied to land-use emissions	No pricing	linearly from 0 in 2020 to 0.5 in 2050	linearly from 0 in 2020 to 0.02 in 2050

In the Low-CDR scenario, we assumed that the global level CCS deployment will not grow to more than 3 GtCO_2_ yr^–1^. This value is consistent with the global deployment of CCS in the High-CDR scenario in 2030. This scenario also assumes higher constraints on biomass feedstock, and availability of ERW by 2050 and considers a limited land carbon price that reaches to 2% of the carbon price imposed on the energy system’s CO_2_ emissions by 2050.

### 2.2 COBRA Module

#### 2.2.1 Dispersion Modeling and Source–Receptor (S–R) Matrix

COBRA version 5.1.5^67^ applies a source–receptor matrix developed by EPA, using the Comprehensive Air Quality Model with Extensions (CAMx).^82^ The matrix is developed by using the source apportionment feature, which tracks the contribution of air pollutant emissions at sources to concentrations at receptors. The CAMx model simulates air quality over many geographic scales. The model treats a wide variety of inert and chemically active pollutants, including O_3_, inorganic and organic PM_2.5_/PM_10_, and mercury and other toxics.

COBRA employs the source–receptor matrix^83^ to translate county-level emissions of primary PM_2.5_, NO_*x*_, SO_2_, and NMVOC into changes in ambient PM_2.5_ and O_3_ concentrations across all U.S. counties. The fixed transfer coefficients in this matrix quantify how a unit release in any source county affects pollutant levels in every receptor county.

Each U.S. county’s emissions of key precursors (primary PM_2.5_, SO_2_, NO_*x*_, NMVOCs) were used as inputs for CAMx with detailed meteorology and chemistry, and the resulting annual average concentration response in every downwind county. The S–R matrix provides a linear transfer coefficient, β_ij_, which quantifies the change in pollutant concentration in receptor county i per unit emission in source county j. In COBRA’s runtime, county-level emissions inventories are multiplied by their corresponding β_ij_ values and summed over all j to yield the net ΔPM_2.5_ and ΔO_3_ in each i. This approach captures both local dispersion and long-range transport and chemical transformation, albeit in a time-averaged, linearized form, enabling more efficient scenario analysis. The description of processing GCAM emission projections to feed in COBRA is provided in SI section S.1.H.

#### 2.2.2 Health Impact Assessment

Health impacts were estimated using COBRA, which incorporates concentration–response functions drawn from peer-reviewed epidemiological literature to estimate avoided premature deaths from long-term exposure to PM_2.5_, short- and long-term O_3_ exposure, as well as reductions in a suite of nonfatal end points. These avoided health end points are then monetized using standard economic valuations: the value of a statistical life for mortality, and cost-of-illness plus willingness-to-pay estimates for morbidity outcomes. The sum of these monetized benefits constitutes COBRA’s total health benefit estimate for each scenario. PM- and O_3_-related health outcomes included in our analysis are provided in SI Table S.5.

COBRA estimates a range for each health outcome by propagating uncertainty from two principal sources: the 95% confidence intervals of epidemiologically derived concentration–response functions for PM_2.5_ and O_3_ (derived from multiple peer-reviewed studies), and the lower and upper bounds of the value of a statistical life (VSL) as recommended by U.S. EPA and U.S. OMB.

### 2.3 FaIR Module

FaIR is a reduced-complexity, computationally efficient climate model that emulates the key physics of the carbon cycle and atmosphere–ocean energy balance. Its capabilities include gas-cycle emulation, global radiative forcing calculation, temperature response, and probabilistic projection. We use FaIR v1.6.2, calibrated to the AR6 Working Group 1 climate assessment.^81,84,85^ The model represents carbon dynamics using four boxes for fast and slow reservoirs, allowing emissions of CO_2_, CH_4_, N_2_O, and other well-mixed gases to be translated into atmospheric concentration pathways. FaIR computes radiative forcings from GHGs, aerosols, and surface albedo changes through lookup tables and analytic functions calibrated against comprehensive climate models. A two-layer energy-balance scheme converts net forcing into global-mean surface temperature and ocean heat uptake, capturing both transient and equilibrium responses. By sampling uncertainty ranges for key parameters—such as transient climate response, gas lifetimes, and efficacy factors—FaIR produces ensembles of warming trajectories with quantifiable confidence intervals.

We adjust FaIR’s SSP2 emissions inputs from 2015 onward using GCAM outputs for fossil-fuel and land-use CO_2_ emissions, as well as for non-CO_2_ species. We also include long-lived fluorinated gases (e.g., CF_4_, C_2_F_6_, HFCs, and SF_6_). FaIR then converts these emissions into atmospheric concentrations, radiative forcings, and global mean temperature change.

### 2.4 Avoided U.S. Climate Damages

The social cost of GHG (SC-GHG) quantifies the net societal harm caused by emitting one metric ton of a given greenhouse gas in a specific year. The carbon price represents the marginal abatement cost required to achieve net-zero emissions at least cost. It is not interpreted as the social cost of carbon or as a direct measure of climate damages. Importantly, our modeling framework does not use U.S.-specific SC-CO_2_ as carbon price, because GCAM solves for prices required to meet CO_2_ emissions constraints exogenously; therefore, in this analysis marginal cost of abatement does not equal marginal climate damages. To calculate avoided climate damages we use SC-GHG values and GHG emissions projections from GCAM. We use EPA’s recently developed SC-GHG to estimate avoided climate damages under High- and Low-CDR scenarios versus NCA-USA baseline for the U.S. Table 3 provides the estimates of social costs for CO_2_, CH_4_, and N_2_O used in our analysis.

**Table 3:** U.S.-Specific SC-GHG from 2025 to 2050 Based on a 2% Near-Term Ramsey Discount Rate, Scaled over Time at 1.5% per Year from 2030

	SC-CO_2_ (USD2020 per metric ton of CO_2_)		SC-CH_4_ (USD2020 per metric ton of CH_4_)		SC-N_2_O (USD2020 per metric ton of N_2_O)	
Emission year	Low	High	Low	High	Low	High
2025	28.8	78.9	436	1114	9002	24128
2030	31	85	470	1200	9700	26000
2035	33.4	91.6	506	1293	10427	28002
2040	36	98.6	546	1393	11258	30176
2045	38.8	106.3	588	1500	12125	32500
2050	41.7	114.4	632	1615	13066	35000

These estimates incorporate the latest scientific findings on climate change and its economic consequences. The SC-GHG enables us to account for the overall social benefits of reducing GHG emissions for use in benefit/cost comparisons. Conceptually, the SC-GHG captures the present value of every future climate impact, capturing impacts to human health, net agricultural productivity, energy systems, buildings and infrastructure, labor productivity, and ecosystem services.^65^ The authors emphasize that these estimates remain partial, as they do not yet include all possible climate pathways and feedbacks (e.g., tipping points, nonmarket ecosystem losses). The full list of climate and earth science impacts and associated damages partially or explicitly included in the estimations is provided in EPA’s working paper on SC-GHG.^65^

The provided SC-GHG is for the year 2030 (SC-CO_2_ = $31–85 USD2020/t, SC-CH_4_ = $470–1,200 USD2020/t, SC-N_2_O = $9,700–26,000 USD2020/t).^65^ We used an escalation factor of 1.5% per year, based on EPA’s global level SC-GHG estimations, to estimate the U.S. national level SC-GHG for 2025 to 2050.

We estimate the undiscounted, monetized value of future emissions changes by multiplying the GHG emission changes of High- and Low-CDR scenarios relative to the NCA-USA in the U.S. by the SC-GHG by period to give the monetized value of future emission changes in each period. The undiscounted monetized value is then discounted back to the present value year (2025) to obtain the present value of the avoided U.S. climate damages.

## 3 Results

### 3.1 Climate Mitigation and Implications for the U.S

Figure 1 presents U.S. CO_2_ emission trajectories (1a), primary energy consumption (1b), and maps of net CO_2_ emissions under the two U.S. net-zero CO_2_ emissions scenarios (1c), High-CDR (1.36 GtCO_2_ yr^–1^ removal in 2050) and Low-CDR (0.41 GtCO_2_ yr^–1^ removal in 2050).

**Figure 1:**
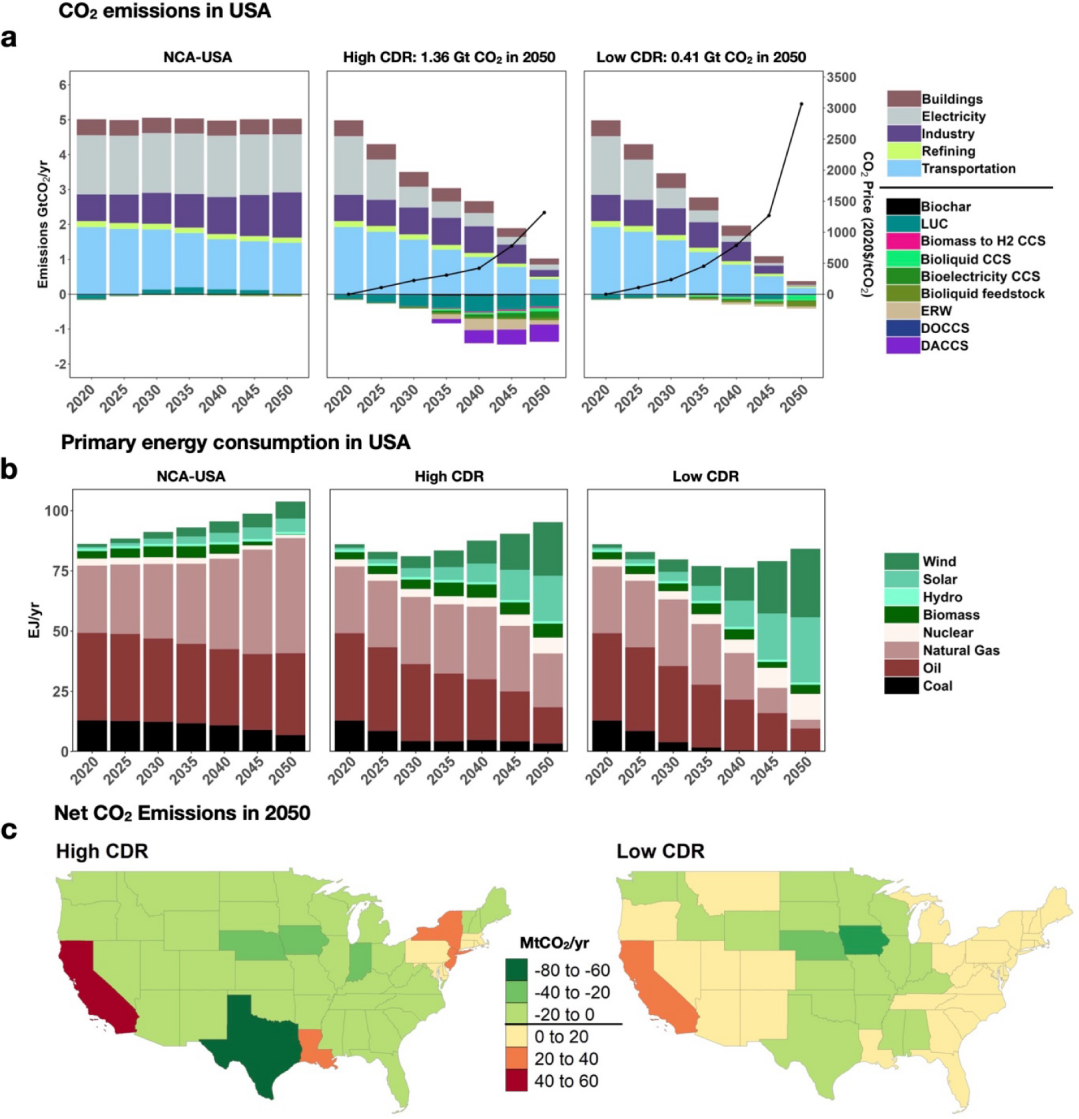
High- and Low-CDR scenarios under U.S. net-zero CO_2_ emissions by 2050 and their implications, analyzed relative to the NCA-USA scenario. **a**. CO_2_ emissions and CDR in the U.S. by 2050. The second *y*-axis represents CO_2_ prices plotted with a black line. **b**. Primary energy consumption by scenario in the U.S. **c**. Net CO_2_ emissions in 2050. The different colors in panel (a) represent the breakdown of U.S. CO_2_ emissions, either positive from the economic sectors (e.g., buildings and electricity) or negative from CDR technologies, in panel (b) the red and black tones represent fossil fuels while the green tones represent renewable energy, and in panel (c) the redder states have net positive emissions while the greener states have net negative emissions.

In NCA-USA, emissions remain near 5 GtCO_2_ yr^–1^ in the U.S. through mid-century, with modest declines in transportation and electricity offset by industrial increases. In High-CDR, residual 2050 emissions fall to 1.03 GtCO_2_ yr^–1^, balanced by a diverse mix of negative emissions technologies. High-CDR projects that 1.85 GtCO_2_ yr^–1^ will be stored in offshore and onshore geological reservoirs in 2050, compared to only 0.23 GtCO_2_ yr^–1^ in Low-CDR scenario. Under Low-CDR, U.S. emissions decline more rapidly because achieving net zero by mid-century requires deeper abatement across sectors without extensive CDR deployment (Figure 1a). Two distinct phases emerge, a gradual decline in emissions through 2045 followed by a sharp reduction toward mid-century. The CO_2_ price rises steeply after 2040 to over $3,000 t^–1^ CO_2_, roughly twice that of the High-CDR case.

In the High-CDR scenario, primary energy demand grows modestly to 95 EJ yr^–1^ by 2050, while in the Low-CDR scenario total primary energy demand falls to 85 EJ yr^–1^. This is because the Low-CDR pathway relies more heavily on fossil emissions reductions through accelerated retirement of fossil fuel infrastructure, rapid electrification, and energy efficiency improvements across industry, buildings, and transportation. These structural shifts induced by the Low-CDR policy design reduce energy demand by shifting away from fossil-based technologies toward more efficient technologies with lower primary energy requirements.

Figure 1c shows maps revealing substantial spatial variation in net emissions, driven by regional differences in e.g., biomass availability, CO_2_ storage capacities, and cropland suitability for ERW. Under Low-CDR, these spatial disparities narrow as limited CCS and CDR lead to broader deployment of renewable energy and fewer regions achieving deep net-negative emissions.

### 3.2 CDR Net Economic Returns Should Not Be Overlooked

Figure 2 shows the regional economic implications of CDR investments by 2050. Figure 2a highlights pronounced difference in cumulative carbon removal achieved through a portfolio of novel CDR technologies across the U.S. In some regions, cumulative removal exceeds 2.5 GtCO_2_ between 2025 and 2050, while several other regions demonstrate capacities greater than 0.5 GtCO_2_ by mid-century, reflecting differences in resource availability, infrastructure, and technology deployment potential.

**Figure 2:**
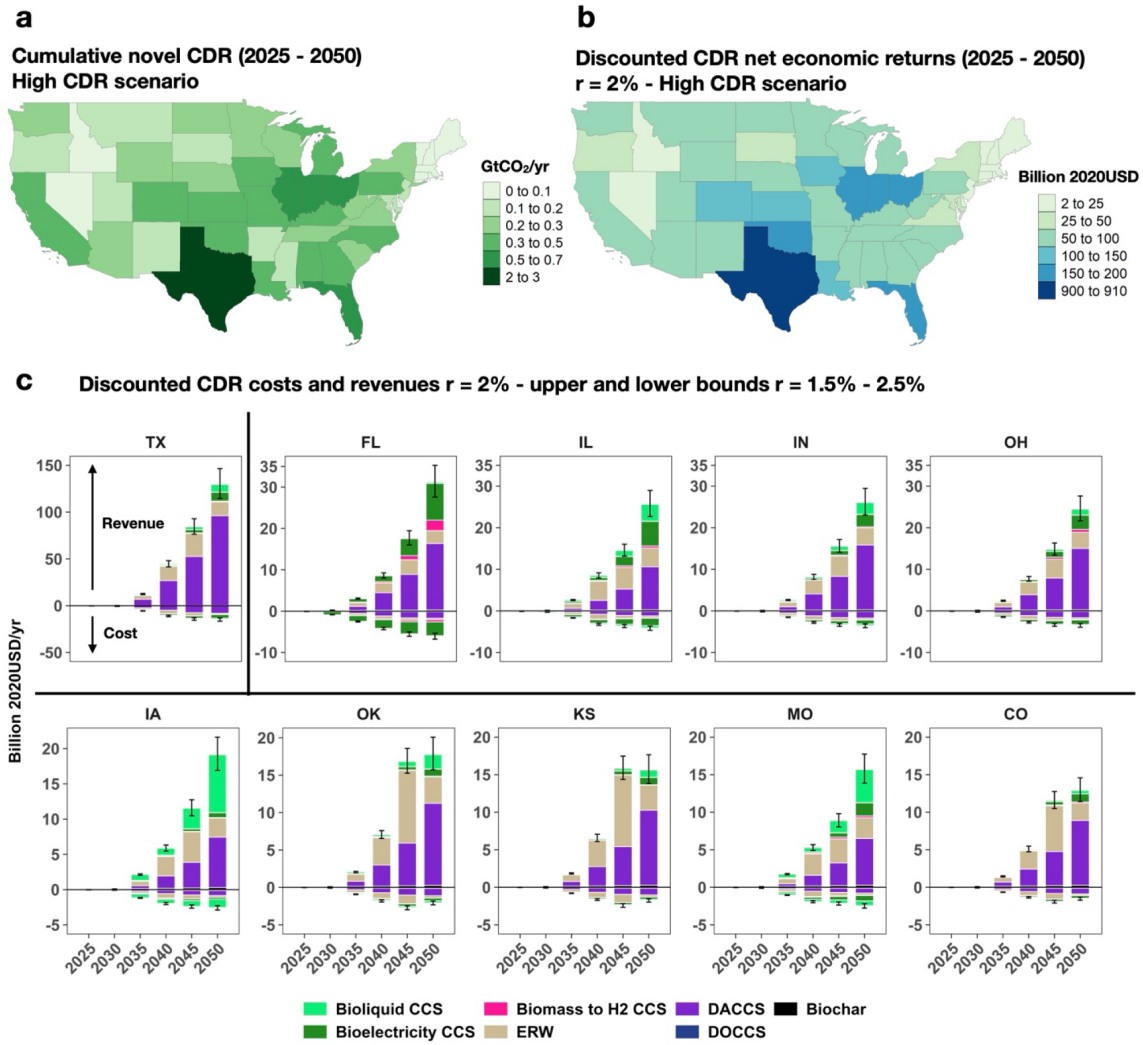
Deployment scale of novel CDR, and their costs and revenues in the High-CDR scenario. **a**. Regional cumulative scale of novel CDR (DACCS, DOCCS, ERW, and BECCS; BECCS includes bioliquid CCS, bioelectricity CCS, and biomass to H_2_ with CCS) from 2025 to 2050. **b**. Discounted CDR net economic returns (2025–2050) with a 2% discount rate. **c.** Discounted CDR costs and revenues in the ten states with the highest CDR revenues in 2050. The main results use a 2% discount rate; upper and lower bounds are calculated using discount rates from 1.5% to 2.5%. Panel (c) is partitioned into three categories with different *y*-axis scales. The revenues do not include the revenues earned from selling bioenergy and biochar.

The discounted CDR net economic returns, which are the regional difference between CDR revenues and costs (Figure 2b), follow a pattern that is similar to the cumulative removal by the states (Figure 2a). Regions with the greatest removal potential generate the highest economic returns, in some states exceeding $900 billion and $150–200 billion USD2020 by 2050 cumulatively (Figure 2b). These economic returns are equivalent to 0.9% and 0.5% of cumulative GDP (CGDP) in Texas and Florida with a 2% discount rate (2025–2050).

Figure 2c presents CDR costs and revenues in regions with the highest revenues. CDR revenues begin emerging around 2035, though areas with greater resource endowment invest earlier and yield the largest gains, reaching $120–150 billion USD2020 by 2050. Much of this revenue derives from DACCS in regions with substantial geological storage potential. Comparing costs to revenues of each technology, ERW can provide a high revenue flow with low costs (Figure 2c).

Large-scale CDR deployment can generate high net economic returns in regions with favorable resource and infrastructure conditions. State-level differences primarily reflect variations in suitable cropland availability for ERW, bioenergy resource endowment, geological CO_2_ storage availability, and the pace of sectoral decarbonization to reach net zero by 2050 that influences CDR deployment over time.

### 3.3 Reliance on CDR Technologies Can Indirectly Increase Air Pollutants Emissions

Policy design plays a key role in the scale of CDR as well as GHG and non-GHG emissions reductions. Coordinated mitigation and CDR strategies can accelerate reaching net-zero emissions, constrain climate overshoot, and provide the highest cobenefits in terms of air quality and public health. In this analysis CDR substitutes for gross emission reductions under a fixed net-zero CO_2_ emissions constraint. Under policy designs with separate targets for sectoral gross emissions reductions and CO_2_ removal, CDR would complement mitigation rather than displace it and would not necessarily increase air pollution.

Sectoral shifts in emissions are indicative of technological and fuel changes in the economic sectors. Under the NCA-USA baseline (Figure 3a), industry, fossil resource production, and transportation collectively account for most NMVOC emissions. NO_*x*_ emissions decline to 7.3 Mt yr^–1^ in 2050 driven primarily by gradual increases in market share for electricity and hydrogen in the transportation sector, and by the transition away from coal power plants. While overall PM_2.5_ and SO_2_ emissions decrease, the industrial sector grows significantly due to expansion in industrial energy use, spanning a diverse set of applications; boilers, process heat, internal combustion engines, as well as specialized processes such as iron and steel, chemicals, aluminum, and other manufacturing facilities.

**Figure 3:**
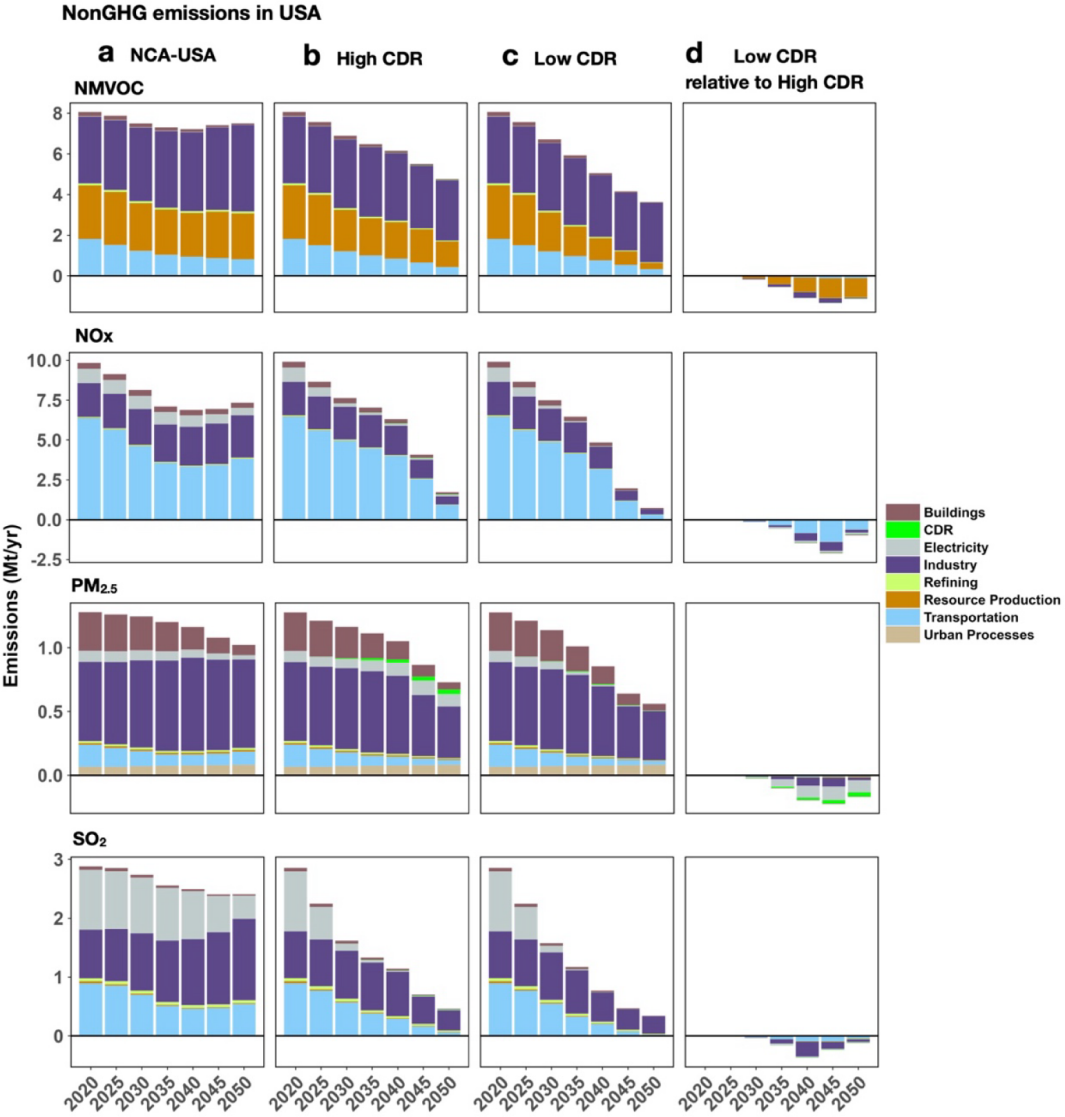
Economy-wide emissions of key air pollutants. Emissions are portrayed for nonmethane volatile organic compounds (NMVOC), nitrogen oxides (NO*_x_*), fine particulate matter (PM_2.5_), and sulfur dioxide (SO_2_).

The High-CDR scenario yields major reductions in U.S. emissions relative to NCA-USA (Figure 3b). Decarbonization generates well understood cobenefits in air quality, and those overall benefits are only slightly affected directly by the CDR technologies.^4,31,86–91^ PM_2.5_ emissions fall by ∼40% to 0.8 Mt yr^–1^, with CDR contributing about 0.036 Mt yr^–1^. NMVOC declines by ∼30% to 5 Mt yr^–1^ as energy systems and industry decarbonize; NO_*x*_ drops to 1.8 Mt yr^–1^ owing to technological changes, such as the transition away from unabated coal and gas, an increase in hybrid and electric market shares for passenger and freight transportation. CCS enables continued fossil powerplant operation, raising PM_2.5_ from electricity generation, while SO_2_ declines from 2.8 to <0.5 Mt yr^–1^, driven by structural changes in energy, transportation, and industrial sectors, such as the retirement of coal powerplants and decreased industrial use of coal.

Low-CDR (Figure 3c) results in lower emissions for all air pollutants. By 2050, NMVOC emissions reach 3.9 Mt yr^–1^, NO_*x*_ near 0.7 Mt yr^–1^, PM_2.5_ around 0.6 Mt yr^–1^, and SO_2_ about 0.4 Mt yr^–1^. Low-CDR incorporates similar mitigation measures that lead to the greatest reductions in High-CDR (e.g., coal retirement and fleet electrification); but in Low-CDR, more reliance on renewable electricity and electrification and less on CCS and CDR has a net effect of reducing air pollutant emissions overall.

### 3.4 CDR Deployment Influences the PM_2.5_- and O_3_-Related Health Cobenefits

Both decarbonization scenarios produce large nationwide reductions in PM_2.5_ and O_3_ concentration relative to NCA-USA, resulting in tens of thousands of avoided premature deaths and hundreds of billions of dollars in health benefits by 2050. These benefits arise from reductions in mortality and morbidity linked to changes in ambient PM_2.5_ and O_3_ concentrations (Figure 4). The full list of health outcomes included in the monetized health benefits is provided in Section 2.2.

**Figure 4:**
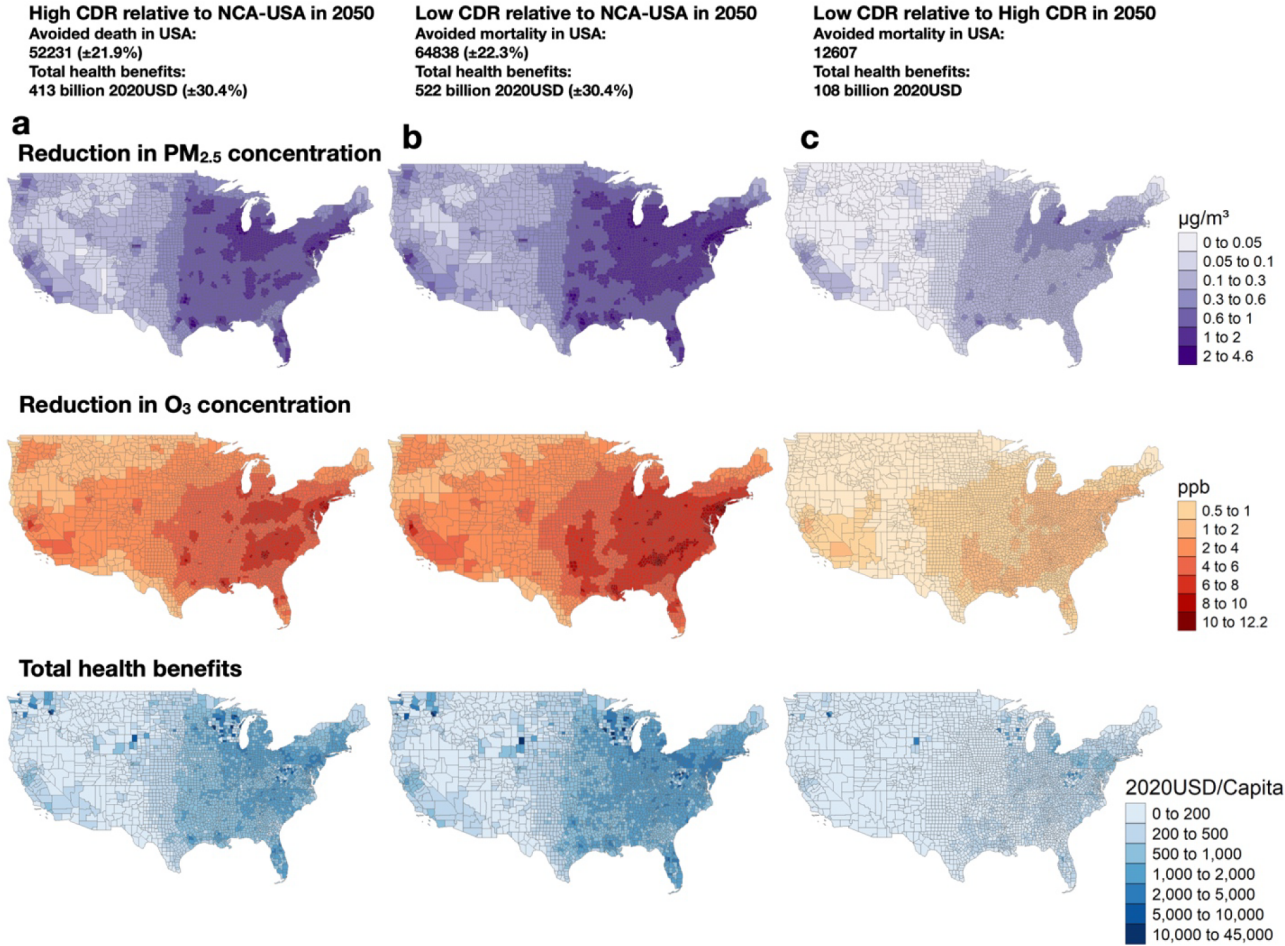
Air quality and public health impacts across the U.S. in **a**, High-CDR and **b**, Low-CDR scenarios both relative to the NCA-USA. Panel **c** demonstrates the additional concentration reductions and public health improvements in Low-CDR relative to High-CDR. Total health benefits are discounted to 2025 value with a 2% discount rate based on guidance from the U.S. Office of Management and Budget (OMB).^95^ COBRA translates air pollutant emissions into ambient concentrations of O_3_ and PM_2.5_. PM_2.5_ concentrations include directly emitted PM_2.5_ and secondary formation from NO*_x_* and SO_2_.

Reductions in PM_2.5_ concentrations are most pronounced in the industrial Midwest and densely populated Northeast, where annual PM_2.5_ levels fall by up to 4.6 μg m^–3^ in 2050 under our decarbonization scenarios relative to NCA-USA. Western states see more modest improvements, typically under 1 μg m^–3^, reflecting their lower baseline pollution and limited transfer of pollutants downwind to the eastern regions.^92^ Declines in O_3_ concentrations are greatest in the Southeast, with the largest concentration drops reaching about 6 ppb in some metropolitan areas but mostly in rural regions. This happens because elevated NO_*x*_ and NMVOC emissions in urban areas are the precursors for ground-level O_3_ formation through photochemical transformations under sunlight and heat. Often, these precursors are transported by prevailing winds, resulting in O_3_ formation in downwind rural regions.^93^

When compared to NCA-USA, the High-CDR avoids over 52,000 premature deaths in the US in 2050 and generates $413.5 billion USD2020 yr^–1^ health benefits. The Low-CDR scenario delivers greater public health improvements by reducing residual emissions, and hence larger reductions in PM_2.5_ and O_3_ precursors, preventing roughly 65,000 premature deaths and generating $522 billion USD2020 yr^–1^ in discounted health benefits compared to NCA-USA in 2050. The largest per capita health gains occur in regions where air quality improves substantially relative to baseline exposure, particularly where populations are older or more sensitive to air pollution–related illness.

On average, Low-CDR can prevent 24% more premature deaths and provide 26% higher health benefits in 2050 than High-CDR. In Low-CDR relative to the NCA-USA, the biggest air-quality improvements, and thus health gains, occur in the industrial Midwest and along the East Coast states, where heavy industry and transportation emissions drive high baseline pollution.^94^

### 3.5 Lower Reliance on CDR Can Significantly Increase the CO_2_ Mitigation Cost

Figure 5a presents cumulative discounted U.S. mitigation costs net of the CDR producer surplus.[Fn fn1] (2025–2050, 2% discount rate) for the High- and Low-CDR scenarios. Under High-CDR, total cumulative discounted mitigation costs are highest in regions with large populations, emissions, and CO_2_ removal potential, reaching at maximum $0.94 trillion USD2020. In Low-CDR, the greater reliance on direct sectoral CO_2_ abatement increases overall costs; thus, the largest CO_2_ emitting regions experience higher mitigation expenditures. Accounting for mitigation cost as a fraction of GDP by 2050, smaller economies bear the highest economic burdens, with several regions exceeding 9% (see SI section S.2.E).

**Figure 5:**
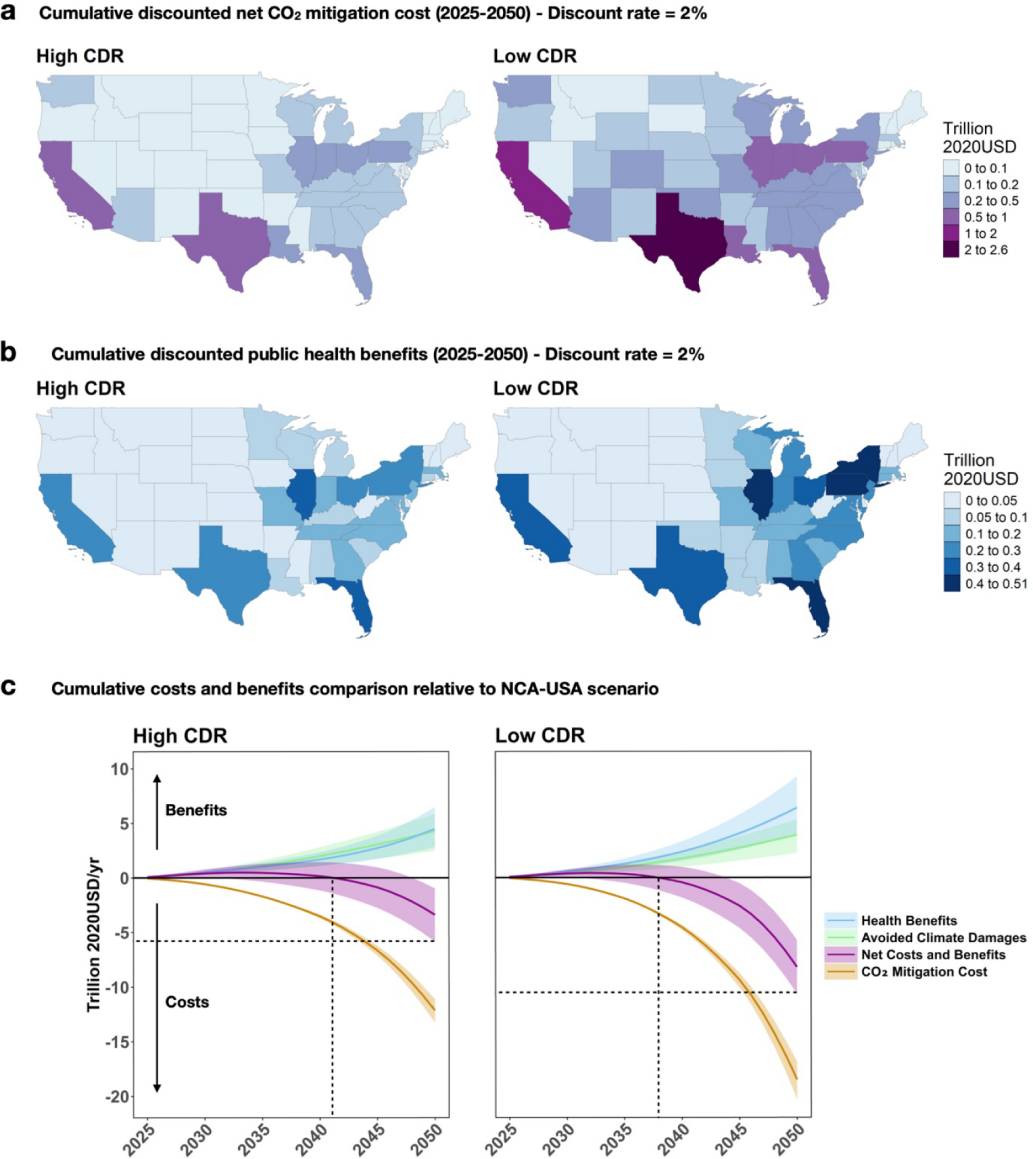
Costs and benefits comparison in the U.S. **a.** Cumulative discounted CO_2_ mitigation cost net of the CDR producer surplus with a discount rate of 2% (2025–2050). **b**. Cumulative discounted public health benefits relative to NCA-USA with a discount rate of 2% (2025–2050). **c**. Comparison of cumulative (2025–2050) avoided U.S. climate damages, CO_2_ mitigation costs, health cobenefits, and net impacts in the U.S., indexed relative to NCA-USA. The upper and lower bounds (shaded regions) for avoided climate damages stem from the considered range for SC-GHG values as well as varied discount rates of 1.5%, 2%, and 2.5% for avoided climate damages calculations. The estimated health benefits are uncertain as well, derived from varying epidemiological studies that assess the impact of PM_2.5_ and O_3_ on health outcomes and varied discount rates of 1.5%, 2%, and 2.5% for the health benefits calculations. The upper and lower bound for CO_2_ mitigation cost stem from varied discount rates of 1.5%, 2%, and 2.5%.

Under High-CDR, states with larger populations face larger public health benefits cumulatively by 2050 (Figure 5b). In the states with the least public health benefits, the baseline emissions and pollutant burdens are comparatively low, and fewer people are exposed, limiting the health benefits achieved because of decarbonization. By contrast, in states with large industrial emissions, dense populations, and greater potential for copollutant reductions, even the same relative emission cuts translate into far greater absolute public health gains. Under Low-CDR, health benefits intensify across the regions, where several populous states gain $0.4–0.51 trillion USD2020 by 2050. Our economy-wide decarbonization yields large cumulative health benefits of $2.8–6.5 (or 0.5–0.9% of CGDP) and $3.5–8 trillion USD2020 (0.6–1.2% of CGDP) by 2050 under High- and Low-CDR scenarios, respectively. These values exceed $1 trillion USD2020 in avoided mortality estimated by Dedoussi et al. (2019) for the U.S. power sector (2002–2017), which was based on PM_2.5_-related mortality from copollutants (SO_2_, NO_*x*_, PM_2.5_) monetized using VSL per unit CO_2_.^96^ A key distinction is that their study period captures primary energy shifting from coal to natural gas, which leads to large reductions in air pollutants emissions, whereas our scenarios involve transitions away from lower-emitting fuels (e.g., natural gas and refined liquids) toward renewables and CO_2_ emissions reduction from harder-to-abate sectors, which generally yield smaller marginal air quality improvements per unit CO_2_ reduced. However, air quality improvement and public health benefits do not scale linearly with broader decarbonization because economy-wide decarbonization introduces trade-offs. Although some CDR would be necessary to offset residual emissions, their operation can contribute to air pollution. In addition, biomass use as primary energy source emits air pollutants, so extending mitigation beyond the power sector can result in smaller marginal air-quality gains per unit of CO_2_ reduced.

In High-CDR, discounted avoided U.S. climate damages grow to $0.4 trillion USD2020 yr^–1^ in 2050, accounting for 1.6% of the national GDP (Figure 5c). Discounted costs of CO_2_ mitigation increase in magnitude to $1.3 trillion USD2020 yr^–1^ (5.3% of the national GDP) in 2050, outpacing damages at mid-century, while health benefits from reduced air pollution rise to $0.68 trillion USD2020 yr^–1^ equivalent to 2.8% of the national GDP in 2050. Under Low-CDR, avoided climate damages drop to $0.4 trillion USD2020 yr^–1^ in 2050. Mitigation costs escalate more steeply, to nearly $2.2 trillion USD2020 yr^–1^ in 2050, 8.9% of the national GDP, driven by more expensive economy-wide structural change with the reduced availability of CDR and CCS. The rapid increase in mitigation costs after 2040 reflects the exhaustion of low-cost abatement options and the need to eliminate challenging residual emissions to reach net-zero emissions in 2050, which sharply drives rising marginal abatement costs. Health benefits expand to $0.8 trillion USD2020 yr^–1^, 3.2% of the national GDP, exceeding those in High-CDR by mid-century as deeper decarbonization leading to more improvements in regional air quality.

When integrating these costs and benefits over time, the net balance of discounted climate and health benefits minus mitigation costs, we can see that the mean net benefits of CO_2_ control in High-CDR become negative around 2041, with a cumulative discounted net present value between −$1.9 and +$1.3 trillion USD2020, or between −0.3% and +0.2% of the national CGDP. In contrast, in Low-CDR, this inflection occurs earlier, around 2037, and cumulative net present value falls to between −$5.7 and −$10.6 trillion USD2020, or between −0.8% and −1.7% of the national CGDP, in 2050. Thus, while both strategies ultimately provide substantial climate and public health benefits, pathways relying more heavily on sectoral decarbonization due to constrained CDR deployment impose considerably higher near- and long-term economic burdens. From a policy perspective, these economic burdens could be reduced through alternative policy architectures that combine binding gross emissions constraints with explicit CDR targets, more flexible timelines toward net-zero emissions, early investment in clean energy, and rapid electrification to accelerate technological learning and cost declines, as emphasized in the broader climate policy literature.^17,47^

## 4 Discussion

This study examines the impacts that CDR technologies have on decarbonization pathways, focusing on regional air quality improvements, global warming mitigation, climate damage reduction, revenues from CDR projects, and the distribution of CO_2_ mitigation costs across national and regional scales. Our analysis carries several caveats. First, GCAM’s state-level emissions are downscaled to county-levels using the current national emissions inventory (NEI) for the U.S., ignoring potential changes in spatial distribution.^2^ Retirement and citing decisions are complex, and may involve factors that are not considered here, such as zoning, permitting, and emission control requirements in areas that already have poor air quality and public opposition.^5^ Translating emission changes to air quality and health impacts also introduces uncertainty by representing complex, atmospheric transport and chemistry processes using a source-receptor matrix.^83^ Second, we do not formally characterize and propagate uncertainty in key techno-economic parameters, such as technology costs, deployment rates, and resource potentials, into our results. Future research should also address broader impacts such as ecosystem health, employment, and equity in regional CDR deployment.

Along with China, India, and Europe, the U.S. plays an outsized role in determining mid-century global temperature trajectories (see SI section S.2.A). While U.S.-only action would not by itself meet Paris Agreement global mean temperature targets, ambitious action by the U.S. can meaningfully reduce the long-term global temperature, potentially helping facilitate broader international climate action. Our modeling reveals regional variation in net emissions by 2050. Under High-CDR, areas with abundant bioenergy resources, geological CO_2_ storage, or suitable croplands for ERW achieve deep net-negative emissions, while regions with limited resources or higher residual emissions remain positive. Low-CDR relies more on renewables and electrification but at a higher marginal CO_2_ price of about $3,000 USD2020 t^–1^CO_2_ in 2050.

The national- and state-level implications of climate action (or inaction) will influence regional air quality, and these average changes in criteria air pollutants will not be distributed evenly across the U.S. Although CDR supply chains contribute only a small fraction of criteria air pollutants, thus affecting local air quality in communities proximate to CDR operations, CDR indirectly elevates NMVOC, NO_*x*_, PM_2.5_, and SO_2_ by permitting residual fossil use to persist longer compared to pathways with limited removal. Under Low-CDR, deeper cuts in residual emissions yield 26% greater health benefits in 2050, avoiding an average of 12,600 more premature deaths in 2050 and saving an average of $108 billion USD2020 in monetized cobenefits from 2025 to 2050, through reduced population exposure to PM_2.5_ and O_3_.

Overall cumulative CO_2_ mitigation costs can be about $5–7 trillion USD2020 lower in High-CDR relative to Low-CDR, yielding net economic savings that accounts for 0.9% to 1.1% of the national CGDP. States with the largest removal and mitigation potential face the highest net mitigation costs (not including the CDR producer surplus) but spend only 0.6–1.5% of their discounted GDP under the High-CDR scenario. In Low-CDR, mitigation costs rise because constrained removal capacity can drive high costs in carbon-intensive and fossil fuel dependent regions. The High-CDR scenario also provides a large revenue flow among the U.S. states, which can reach $1 trillion USD2020 in 2050 alone (4% of the national GDP in 2050). The scenarios reveal a clear trade-off between costs and benefits. Greater reliance on CDR can operate as an economy-wide flexibility option that reduces the cost of the final units of abatement from hard-to-abate sectors, which lowers overall mitigation costs but yields less public health benefits, whereas deeper sectoral decarbonization under Low-CDR delivers larger public health benefits at substantially higher mitigation costs. A uniform national policy approach for all the states might exacerbate regional inequities in terms of socioeconomic outcomes unless accompanied by targeted federal incentives, revenue-sharing mechanisms, or transition assistance for jurisdictions that will be most exposed to carbon-constrained economic disruption.

Although relative to NCA-USA the net costs and benefits in the High-CDR is higher than Low-CDR, these results should not be interpreted as evidence that decarbonization is economically unjustified. Two key considerations need to be taken into account. First, the analysis captures only domestic U.S. climate damages, excluding the much larger global benefits of avoided global warming. Because the modeling assumes the rest of the world also decarbonizes, the U.S. would internalize part of those global gains through trade, diplomacy, or collective climate action. The U.S. contribution to global mitigation, therefore, carries benefits that extend well beyond its borders, and the values presented here represent a lower bound on the true climate benefits that would be realized. Second, net-zero by 2050 is a political benchmark rather than an economic optimum. The rapidly rising marginal costs of eliminating the final units of CO_2_ make a slower, more flexible decarbonization trajectory, followed by a period of net-negative removals, more cost-effective in the long run. This is particularly evident in the Low-CDR scenario, where constrained CDR drives up mitigation costs and would likely delay the attainment of net-zero under an economically optimal path. Our analysis, therefore, compares the most cost-effective pathways to a fixed policy target, not the optimal economic schedule for decarbonization.

## Supplementary Material



## Data Availability

GCAM is an open-source model available at https://github.com/JGCRI/gcam-core/releases. The specific version of GCAM, additional input files along with the data processing scripts are available at 10.5281/zenodo.18760906.

## Footnotes

1 This cost figure is not an estimate of the *net* state policy cost, since it does not account for the funds or transfers needed to induce the CDR in each state. For example, under a national climate policy consistent with this scenario, the CDR would receive compensation under a national emission market or a national GHG tax.

## Notes

Additional details on methodology for CDR abatement costs, emission factor modification, non-CO_2_ emissions accounting, CO_2_ mitigation cost calculations, and energy systems projections, as well as supplementary results for global warming impacts, CCS emissions, state-level air pollution, CDR deployment potential, and cumulative mitigation costs (PDF)
