# Supplementary material for: Large-Scale Carbon Removal Will Create Public Health, Economic, and Climate Trade-Offs
Source: Environ Sci Technol. 2026 Jun 8;60(24):17192–205. doi: 10.1021/acs.est.5c17950 (PMC13296501; doi:10.1021/acs.est.5c17950)
Supplement: Supplementary file 1 [file es5c17950_si_001.pdf]

Large-Scale Carbon Removal Will Create Public Health, Economic, and Climate Tradeoffs

Parisa Javadi[1], Patrick O'Rourke[2], Jay Fuhrman[3], Daniel H Loughlin[4], Scott C. Doney[5], William Shobe[6], Joao Ferreira[6], and Andrés F. Clarens[1*]

[1] Department of Civil and Environmental Engineering, University of Virginia, Charlottesville, Virginia, 22904, United States
[2] University of Maryland, College Park, Maryland, 20742, United States
[3] Center for Global Sustainability, University of Maryland, College Park, Maryland, 20742, United States
[4] Nicholas School of the Environment, Duke University, Durham, North Carolina, 27708, United States
[5] Department of Environmental Sciences, University of Virginia, Charlottesville, Virginia, 22904, United States
[6] Batten School of Leadership and Public Policy, University of Virginia, Charlottesville, Virginia, 22904, United States

* Email: andres@virginia.edu

Contents

S.1. Methodology **4**

S.1.A. CDR abatement costs 4

S.1.B. Emission factor modification in GCAM 5

S.1.C. Non-CO_2 emissions accounting for post-combustion CCS and CDR 8

S.1.D. State-level geological carbon storage capacities by state within GCAM-USA 12

S.1.E. CO_2 mitigation cost calculation in GCAM 12

S.1.F. CDR costs and revenues 14

S.1.G. Energy systems and emissions projections 14

S.1.H. Use of GCAM emission projections in COBRA 16

S.2. Additional results **17**

S.2.A. Global warming will be impacted by U.S. climate policy 17

S.2.B. Emissions from electricity generation with CCS 19

S.2.C. State-level criteria air pollution by scenario 20

S.2.D. Marginal abatement cost and deployment potential of CDR technologies in the U.S. under High-CDR scenario 21

S.2.E. Cumulative discounted net CO_2 mitigation costs as a fraction of cumulative discounted GDP 24

References **25**

List of Figures

Figure S.1. State-level abatement cost by novel CDR technologies in 2050 in the U.S. 4

Figure S.2. State-level average wind speed of 40% windiest regions of each state at 10-meter height..................11

Figure S.3. State-level CO_2 storage capacity ($MtCO_2$) extracted from GCAM-USA core model 12

Figure S.4. Conceptual representation of emissions mitigation cost calculation in GCAM. This figure is retrieved from GCAM v8.2 Documentation: GCAM Policies[6] 13

Figure S.5. Change in global radiative forcing and impact on global average temperature change relative to preindustrial (1850–1900) levels. 18

Figure S.6. CO_2 sequestration and associated emissions in electricity sector in 2050 - High CDR........................19

Figure S.7. State-level non-CO_2 emission in 2050 20

Figure S.8. Marginal abatement cost and deployment potential of CDR technologies in Texas under High-CDR scenario 21

Figure S.9. Marginal abatement cost and deployment potential of CDR technologies in Iowa under High-CDR scenario 22

Figure S.10. Marginal abatement cost and deployment potential of CDR technologies in California under High-CDR scenario 23

Figure S.11. Cumulative discounted net CO_2 mitigation costs as a fraction of cumulative discounted GDP (2025-2050) with an interest rate of 2%...24

List of Tables

Table S.1. Emission factors for electricity generation with CCS 5

Table S.2. Emission factors for transportation sector...6

Table S.3. Emission factors for hydrogen combustion..7

Table S.4. Emission factors for bioelectricity generation technologies and the slow pyrolysis process for biochar production 9

Table S.5. PM- and O3-related health outcomes included in this analysis...17

S.1. Methodology

S.1.A. CDR abatement costs

Figure S.1 presents the state-level CO_2 abatement costs in 2050 by the included novel CDR technologies in GCAM-USA.

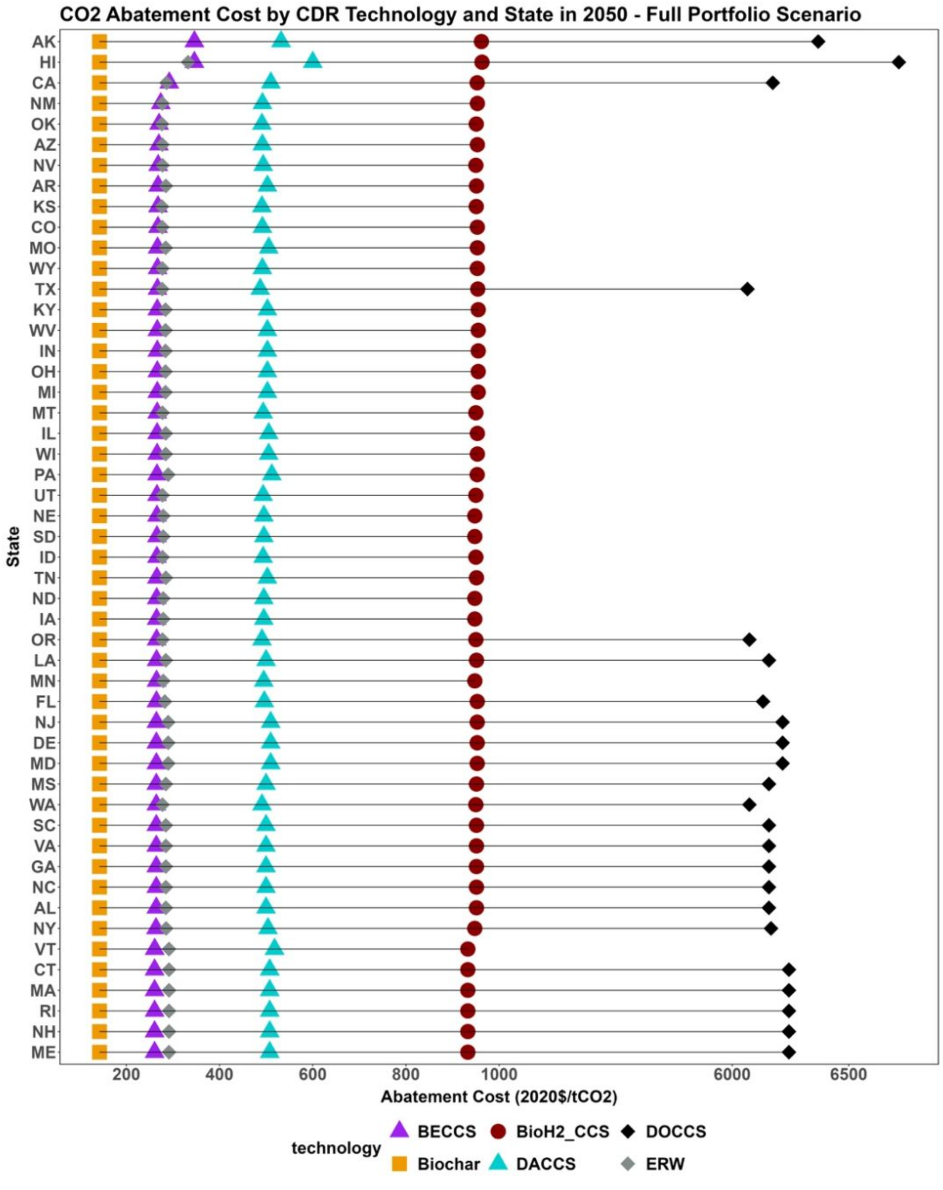

Figure S.1. State-level abatement cost by novel CDR technologies in 2050 in the U.S.

S.1.B. Emission factor modification in GCAM

GCAM's emission factors for electricity generation with CCS are updated based on the values proposed by European Environment Agency's report on air pollution impacts from carbon capture and storage (CCS).[1] Because of the lack of data for all different types of electricity generation with CCS, we used the emission factors for post-combustion carbon capture for pulverized coal with CCS and applied them to other technologies for electricity generation with CCS. Therefore, the modified values are reduced about ~85% for SO_2, ~6% for NO_x, ~30% for $PM_{2.5}$.

Table S.1. Emission factors for electricity generation with CCS

Technology	Category of value	Emission Factors (kg/GJ)			
		SO_2	NOx	$PM_{2.5}$	PM_{10}
Biomass (conv CCS[1])	Original	0.03440	0.00861	0.03110	0.03470
	Modified	0.00516	0.00809	0.02208	0.02464
Biomass (IGCC CCS[2])	Original	0	0.00473	0.00013	0.00013
	Modified	0	0.00445	0.00009	0.00009
Coal (conv pul CCS[3])	Original	0.02580	0.03010	0.01600	0.02130
	Modified	0.00387	0.02829	0.01136	0.01512
Coal (IGCC CCS)	Original	0.00645	0.00559	0.06960	0.23900
	Modified	0.00097	0.00525	0.04942	0.16969
Refined liquids (CC CCS[4])	Original	0.03110	0.24300	0.00623	0.02460
	Modified	0.00467	0.22842	0.00442	0.01747
Gas (CC CCS)	Original	0	0.00473	0.00013	0.00013
	Modified	0	0.00445	0.00009	0.00009

[1]Conversion with CCS
[2]Integrated gasification combined cycle with CCS
[3]Conventional pulverized with CCS
[4]Combined cycle with CCS

Emission factors for transportation using hybrid liquids are included in GCAM-USA as shown in Table S.2. The emission factors are retrieved from European Monitoring and Evaluation Programme (EMEP)/EEA Air Pollutant Emission Inventory Guidebook (2023).[2]

Emission factors for Light truck are based on light commercial vehicle (LCV) category of vehicles that use petrol.

Emission factors for freight rail are based on railways source category that uses gas oil or diesel fuel.

Emission factors for maritime transport are based on national and international navigation source categories that use marine diesel oil or marine gas oil.

Table S.2. Emission factors for transportation sector

**GCAM sector**	**Technology**	**Fuel**	**Emission Factors (kg/GJ)**			
			**SO_2**	**NOx**	**$PM_{2.5}$**	**NMVOC**
**Road transport**	**Light truck**	Hybrid liquids	0.0002	0.3467	0.0353	0.0358
**Freight rail**	**Train**	Hybrid liquids	0.0023	1.2190	0.0319	0.1081
**Marine transport**	**Domestic ship**	Hybrid liquids	0.0434	1.6409	0.1049	0.0406
**Marine transport**	**International ship**	Hybrid liquids	0.0434	1.6409	0.1049	0.0406

Based on guidance from the EMEP/EEA Air Pollutant Emission Inventory Guidebook (2023),[2] hydrogen combustion in stationary gas turbines is expected to emit levels of NO_x comparable to those produced by natural gas under similar operational conditions. Since low-NO_x and aftertreatment technologies are already well established for natural gas systems and can be adapted for hydrogen applications, the guidebook advises using equivalent NO_x emission factors for both fuels. Following this approach, the natural gas NO_x emission factors were applied to all hydrogen combustion activities in GCAM-USA, including process heat in cement manufacturing, backup electricity generation, and other industrial energy uses.

We added NO_x emission factors for aviation based on the guidebook as well. The guidebook recommends scaling factors for hydrogen fueled-aviation relative to kerosene in turbine-powered aircraft derived from a modeling study by Khan et al. (2022).[3] This study found that hydrogen combustion generates substantially lower NO_x emissions during cruise—approximately one order of magnitude lower than conventional jet fuel—but slightly higher emissions during the landing and take-off (LTO) cycle due to higher flame temperatures. We applied a scaling factor of 0.1 to the cruise-phase NO_x emission factors used for conventional domestic and international aviation in GCAM-USA.

Table S.3. Emission factors for hydrogen combustion

GCAM sector	Technology	Fuel	Emission Factors (kg/GJ)			
			SO_2	NOx	$PM_{2.5}$	NMVOC
**Industrial energy use**	**Process heat for cement production**	Hydrogen	0	0.0740	0	0
**Back-up electricity**	**Hydrogen combustion turbine**	Hydrogen	0	0.0740	0	0
**Other industrial energy use**	**Hydrogen combustion**	Hydrogen	0	0.0740	0	0
**Aviation**	**Domestic**	Hydrogen	0	0.0248	0	0
**Aviation**	**International**	Hydrogen	0	0.0274	0	0

S.1.C. Non-CO_2 emissions accounting for post-combustion CCS and CDR

CDR pathways rely on energy-, land- and/or resource-intensive supply chains, which may generate unwanted emissions of NOx, SO_2, NMVOC, $PM_{2.5}$, and other air pollutants at multiple stages, such as from biomass cultivation, feedstock processing, energy consumption to operate the CDR process, and transportation of resources to deliver negative emissions. For CCS and CDR technologies, we adjusted and included emission factors in GCAM-USA based on literature values, EPA reports, and EMEP air pollutant emission inventory guidebooks. The representation of the emissions from CDR technologies reflects technological system boundaries, energy and electricity inputs, and regional operations.

To account for the emissions from electricity generation with post-combustion CCS, we adjusted GCAM-USA's default non-CO_2 emission factors using the values provided in air pollution impacts from CCS technical report of the European Environment Agency.[1] The original and adjusted emission factors are provided in Table S.1.

Emissions from electricity consumption for the operation of DACCS, basalt rock comminution for ERW, bioH_2 with CCS, and electrochemical separation of seawater for DOCCS are calculated endogenously in GCAM by multiplying the activity of each technology by the relevant emission factors, considering the regional electricity mix.

High temperature natural gas based DACCS is defined to use oxy-fuel combustion of natural gas with CCS for process heating, resulting in no co-pollutants. Oxy-fuel combustion is regarded as a clean technology because it uses nearly pure oxygen instead of air, resulting in flue gas that is rich in CO_2. This concentrated CO_2 stream makes capture and storage much more straightforward compared with conventional combustion. Furthermore, the reduced volume of flue gas in oxy-fuel systems simplifies the removal of pollutants such as SO_2 and NO_x.

The slow pyrolysis process for biochar production generates biogenic syngas as a co-product that can be used as a carbon neutral energy source. The process yields 20.1 GJ of syngas co-product and requires 0.2 GJ gas or thermal input per ton of biochar.[6,7] Therefore, the biochar model in GCAM assumes that the thermal input to the pyrolysis process is met by a fraction of the syngas co-product. We exogenously account for the emissions from combustion of syngas to operate the slow pyrolysis facility (provided in Table S.4).

Bioelectricity includes two types of technologies: biomass energy conversion to electricity with carbon capture and storage (conv CCS) and integrated gasification combined cycle of biomass with carbon capture and storage (IGCC CCS) The emission factors for these technologies are provided in Table 2. GCAM includes four types of technologies for bioliquid production with CCS: cellulosic ethanol and Fischer-Tropsch syn-fuels, each with two levels of carbon capture. The first CCS level generally consists of relatively pure and high-concentration CO_2 sources (e.g., from gasifiers or fermenters), which have relatively low capture and compression costs. The second CCS level includes a broader set of carbon sources (e.g., post-combustion emissions) and incurs higher costs but has a higher CO_2 removal fraction. These bioliquid production technologies do not contribute to criteria air pollutants; however, GCAM accounts for CH_4 and N_2O emissions from these technologies.

Table S.4. Emission factors for bioelectricity generation technologies and the slow pyrolysis process for biochar production

CDR technology	Activity	$PM_{2.5}$ (kg/t CO_2 removal)	NOx (kg/t CO_2 removal)	SO_2 (kg/t CO_2 removal)	NMVOC (kg/t CO_2 removal)	Reference
Bioelectricity	Integrated Biomass Gasification Combined Cycle with CCS	0.0015	0.0561	0	0.0030	GCAM data system
	Biomass Conversion with CCS	0.3688	0.1021	0.4079	0.0898	
Biochar	syngas consumption for the slow pyrolysis process	0.2886e-4	0.0027	0.2479e-4	0.0008	[6,8]
ERW	Liquid fuels consumption for mining machinery	0.0013	0.0342	0.0031	0.0017	[8,9]
	Controlled tertiary crushing	1.5119e-4	0			[10]
	Controlled fines crushing	1.0584e-4	0			[10]
	Controlled screening	0.7559e-4	0			[10]
	Controlled conveyor transfer point	0.1965e-4	0			[10]

CO_2 removal by ERW includes the following steps: basalt rock querying and mining, rock crushing and screening, handling and storage of the material, fine rock transportation, and spreading on croplands. Emissions from rock mining stage is exogenously calculated using the emission factors provided in Table S.4. The emission factors translated from grams of pollutant generated per gigajoule of energy (from EMEP[8]) to kilogram of emissions per ton of CO_2 removed by ERW, assuming 0.02 GJ per ton rock is required for mining and 0.3-ton CO_2 gets removed per ton of basalt rock application on croplands[9,11,12] for crushing, screening, and conveying stage.

For the stages that include handling, storage piles, and crushed rock spreading, we consider the methodology of EPA's AP-42, Compilation of Air Pollutant Emissions Factors from Stationary Sources, introduction to fugitive dust sources of chapter 13.[10] Thus, Equation S.1 is used to calculate the emission factor for these stages.

Equation S.1:

$$EF = \frac{k \times 0.0032 \times (\frac{U}{5})^{1.3}}{(\frac{M}{2})^{1.4}}$$

where EF is in lbs. per ton rock, k is a dimensionless particle size multiplier, U is average wind speed (mph) at the site, and M is the material moisture content (%). For particle sizer smaller than 2.5 μm ($PM_{2.5}$) k is equal to 0.053.[4] Applying water and chemical wetting agents are the primary strategies for reducing dust from aggregate storage piles. The default value of M for uncontrolled operations is 0.7% and for controlled operations is 2%.[13] We consider a moisture content of 2% for handling and storage piles and a moisture content of 0.7% for uncontrolled crushed rock spreading. For wind speed in this equation, we used the state-level average wind speed of 40% windiest regions of each state at 10-meter height, retrieved from global wind atlas.[5] The regional wind data is provided in Figure S.2.

We assume an average transport distance of 300 km and 0.3 tCO_2 per ton basalt rock, leading to a freight transport input of 1,000 tonne-km per tCO_2 removal[11,12]. Therefore, emissions from freight transportation of fine rocks for ERW to croplands are calculated endogenously and extracted from total emissions from freight transportation for the states.

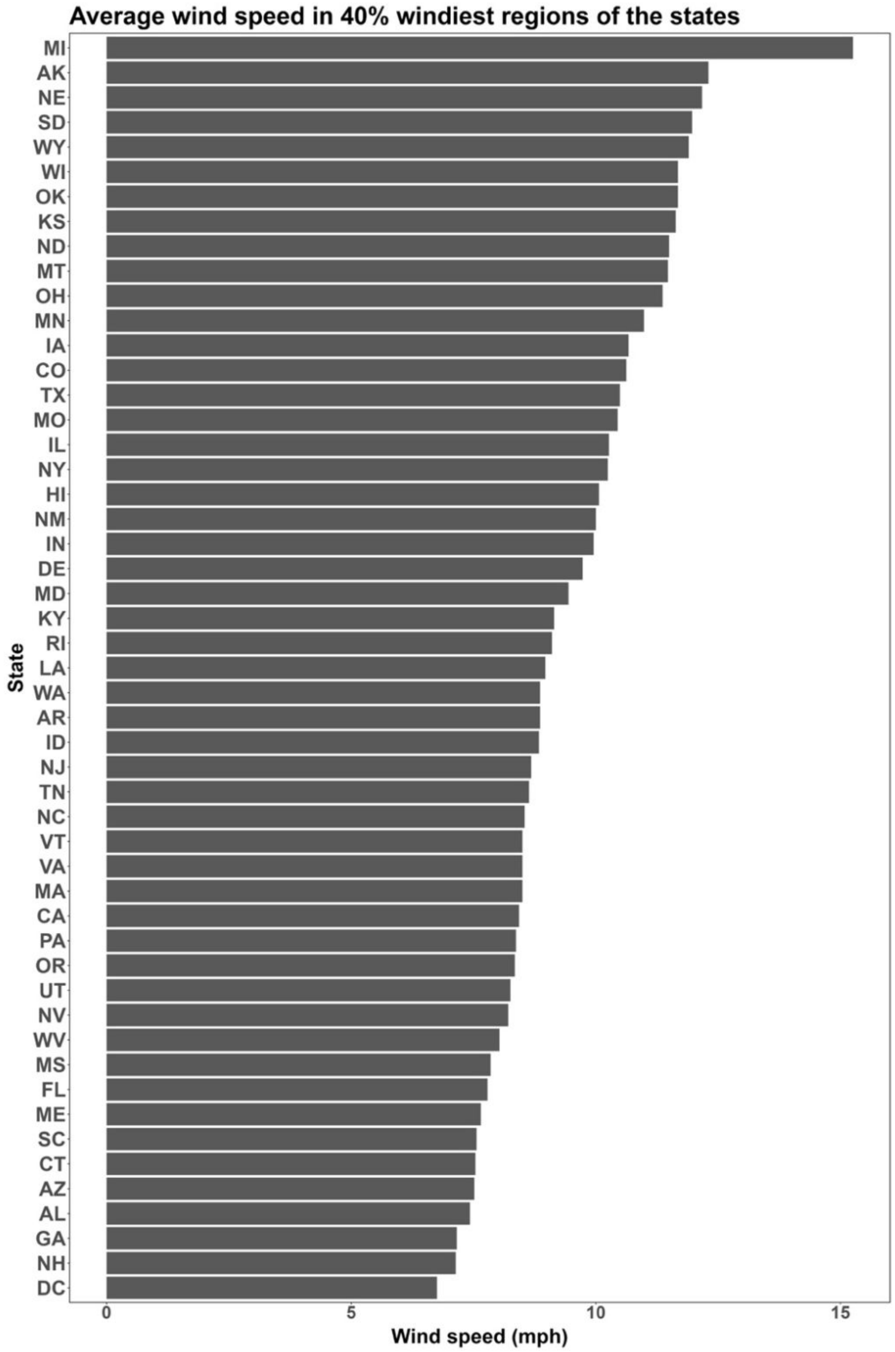

Figure S.2. State-level average wind speed of 40% windiest regions of each state at 10-meter height.

S.1.D. State-level geological carbon storage capacities in GCAM-USA

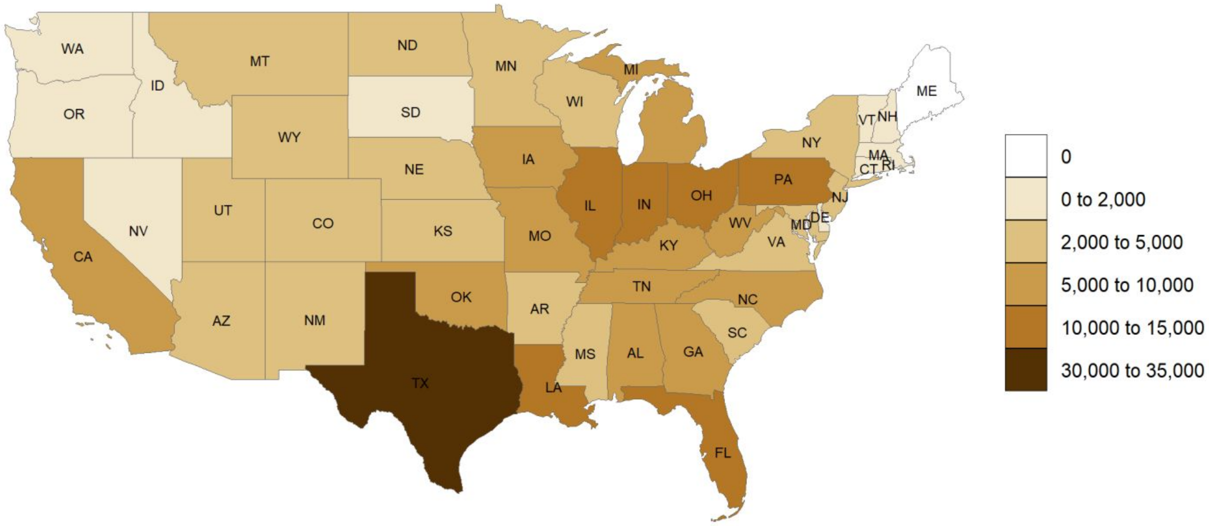

Figure S.3. State-level CO_2 storage capacity ($MtCO_2$) extracted from GCAM-USA core model.

GCAM-USA accounts for 326 distinct geological storage formations across the U.S. Figure S.3 depicts the state-level CO_2 storage capacity, extracted from GCAM-USA core model.
In GCAM-USA, CCS deployment is constrained by regional CO_2 transport and geologic storage cost curves derived from spatially explicit source-sink cost optimization analysis across 326 U.S. geologic storage reservoirs by Dahowski et al (2005, 2010, and 2011).[14–16] These supply curves include site characterization, capital, operations and maintenance, monitoring, verification, and regulatory compliance costs for injection into suitable deep reservoirs, while CO_2 capture, and compression costs are accounted for at the source of capture. Implemented as exhaustible resources, the cost curves preferentially deploy low cost, long-duration storage first and access higher cost options as capacity is depleted, thereby endogenously increasing marginal storage costs over time and shaping the spatial pattern of DACCS deployment. Although DAC facilities themselves are not explicitly sited, storage availability and transport costs favor deployment in regions with large geologic storage potential, consistent with recent high resolution national assessments of U.S. CCS feasibility, in Roads to Removal report and the study by Dai et al (2025).[17,18]

S.1.E. CO_2 mitigation cost calculation in GCAM

To evaluate the effect of high- and low-CDR availability on mitigation costs in the U.S., we estimated discounted CO_2 mitigation costs (2025-2050), including sectoral decarbonization and CDR implementation costs across the states. We used a simplified version of the methodology discussed by Bradley et al.[19] We established a five-point mitigation cost curve representative of discrete carbon pricing intervals (0%, 40%, 60%, 80%, and 100%). This curve facilitated the calculation of the area beneath it, which corresponds to the aggregate mitigation cost in each year from 2025 to 2050. We calculated this area by integrating the product of the shadow price for CO_2 emissions in the net-zero scenarios $P_{S,y}$ and the incremental CO_2 emission reductions at the stipulated carbon pricing points. This computation not only encompassed the costs associated with the shadow price of CO_2 emissions for adhering to the annual emission reduction targets but also consolidated these costs over the 25 years. The analysis was done for

each year within the study's temporal boundary, and a 2% discount rate (r) was applied to normalize future costs to their present value as of the base year 2025. Having the cumulative policy cost C_p, we could quantify the economic implications of achieving net-zero emissions within the specified timeframe, following Equation S.2 to calculate the total mitigation cost C_p.
Equation S.2:

$$C_p = \sum_{y=2025}^{y=2050} \left(\int_{P_{S,y}^{1}}^{P_{S,y}^{5}} P_{S,y} * dE_{S,y} \right) * \frac{1}{(1+r)^{y-2025}}$$

C_p is the cumulative policy cost over the period from 2025 to 2050.
$P_{S,y}$ is the shadow price function for CO_2 emissions at each point within the $dE_{S,y}$ specified carbon pricing intervals in net-zero scenario S for year y.
$dE_{S,y}$ denotes the differential in CO_2 emissions, representing the incremental reduction at each carbon pricing point.
r is the annual discount rate, set at 1.5, 2, and 2.5%, which discounts future mitigation costs back to their present value in the year 2025.
Figure S.4 shows a conceptual representation of emissions mitigation cost calculation in GCAM.[20] The mitigation cost is computed as the triangular area (purple) between the reference emissions path without a carbon price (blue) and the policy-constrained path under a carbon price (green). The shaded area represents the resource cost of successive abatement steps, where each reduction in emissions corresponds to an incrementally higher carbon tax. The final ton of CO_2 abated is equal to the carbon price in that period. The red rectangle indicates the associated tax revenue, calculated as the carbon price multiplied by the remaining emissions. This approach provides a tractable welfare-based estimate of mitigation cost used in GCAM's policy analysis.

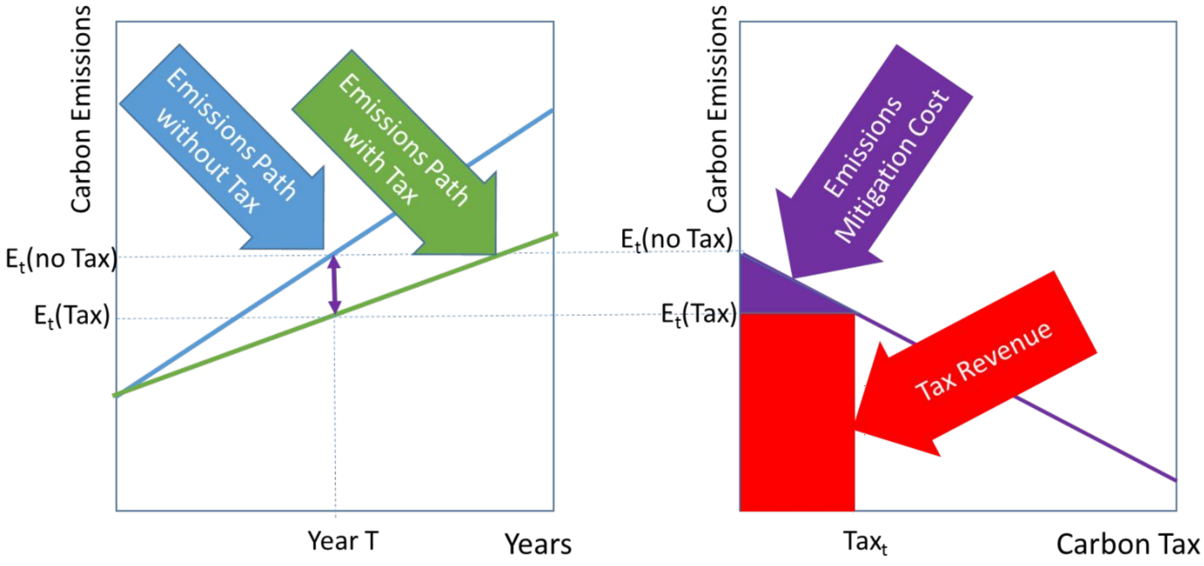

Figure S.4. Conceptual representation of emissions mitigation cost calculation in GCAM. This figure is retrieved from GCAM v8.2 Documentation: GCAM Policies. [20]

S.1.F. CDR costs and revenues

To quantify the economic implications of CDR deployment, we calculated both the total costs and revenues of individual CDR technologies using outputs from GCAM-USA. This analysis is performed on the novel CDR approaches (BECCS, DACCS, ERW, Biochar, and DOCCS) under each scenario's endogenously calculated carbon price. GCAM determines this price based on the CO_2 constraint that equilibrates emissions and removals across the economy, meaning that each technology's cost and deployment are jointly determined by the equilibrium market for CO_2 allowances.

Costs and revenues correspond respectively to the expenditure on production and the producer surplus arising from the policy-driven carbon market. A producer surplus represents the difference between the market price that a producer receives and the marginal cost of production, summed over the total quantity supplied. In the context of CDR, the market price is the model-imposed carbon price, and the marginal cost of production is the technology-specific abatement cost reported by GCAM.

For each technology i in region r and year t, GCAM provides the quantity of CO_2 removed ($Q_{r,i,t}$ $MtCO_2\ yr^{-1}$) and its average abatement cost ($C_{r,i,t}$ 2020\$/tCO_2). The carbon price (P_c) represents the policy incentive level (109–1318 \$/tCO_2 between 2025 and 2050 in the High-CDR scenario). We compute the technology-level investment cost and producer revenue as:

Equation S.3: $Investment_{r,i,t} = C_{r,i,t} \times Q_{r,i,t}$

Equation S.4: $Revenue_{r,i,t} = (P_c - C_{r,i,t}) \times Q_{r,i,t}$

Equation S.3 represents the total resource expenditure required to deploy each CDR technology (capital and operating cost), while equation S.4 captures the positive producer surplus generated when the carbon price exceeds the technology's abatement cost.

Costs and revenues are computed for each U.S. state and CDR technology in GCAM-USA, including BECCS, DACCS, ERW, Biochar, $BioH_2$ with CCS, and DOCCS, and then aggregated to obtain regional and national totals. All monetary values are discounted to present value relative to 2025 using constant discount rates (r) of 1.5, 2, and 2.5%.

This framework is conceptually equivalent to the area between the equilibrium carbon price line and the CDR supply curve in classical microeconomics, where the difference between the CO_2 price and the marginal abatement cost reflects per-ton producer surplus for the CO_2 removed by each technology in each region and period. Aggregating across all technologies yields an estimate of total economic surplus from the deployment of CDR under given policy incentives. More details about the result of this regional analysis are included in section S.2.D.

S.1.G. Energy systems and emissions projections

GCAM is a technology-rich, logit-based, dynamic-recursive model. GCAM simulates the supply and demand across thousands of markets, solving each market by determining the prices that equate supply and demand. For each market, technology shares are determined probabilistically according to their

relative costs in order to simulate the mix of technology sales, commodity demand responses, and the retirement of existing capital stock. The model steps incrementally through time, starting each subsequent period with the technological stock and prices determined from the prior period. Given a particular CO_2 constraint, the model solves for a CO_2 price that satisfies the requirements of the given scenario.

Energy systems and emission projections for the U.S. through 2050 were from GCAM-USA, which applies state-specific projections of population and economic growth, aligned with Shared Socioeconomic Pathway 2[21] to capture regional heterogeneity in labor productivity and demographic trends. Its representation of the energy system simulates supply, conversion, and demand. Energy supplies include fossil fuels such as coal, crude oil, and natural gas, as well as non-fossil energy resources, such as solar, wind, biomass, and uranium. These raw types of energy are converted into useful forms (e.g., in modeled representations of electric power plants, as well as petroleum and biofuel refineries). Useful energy is then transported to meet energy demands within end-use sectors, including in residential and commercial buildings, on road and nonroad transportation, agriculture, and industry.

Within the power sector, GCAM-USA embeds U.S.-specific technology cost trajectories, state-by-state coal and nuclear retirement schedules, and five-year planning increments. It employs fifteen regional load-duration curves, each segmented into baseload, intermediate, sub-peak, and peak periods. Renewable supplies are state-sourced, while fossil fuels clear through a national market. Most technology cost inputs are derived from the National Renewable Energy Laboratory's (NREL) 2019 Annual Technology Baseline,[22] incorporating projected capital and operation and maintenance changes through time.

Final energy service demands, residential and commercial floor space heating and lighting, industrial combustion and process energy (including cement and fertilizer manufacture), and transport in passenger-km and freight-tonne-km, are disaggregated at the state level. The transport module allows for vehicle electrification (cars and trucks), with cost and efficiency parameters guided by NREL's Electrification Futures Study.[23]

GCAM quantifies emissions of major greenhouse gases (CO_2, CH_4, N_2O, HFCs, PFCs, SF_6) using data from the Community Emissions Data System (CEDS).[24] Because CEDS excludes biomass burning and land-use change, emissions from grassland and forest fires, deforestation, and residue burning are supplemented with the Global Fire Emissions Database (GFED).[25] Mode-specific road-transport emissions are derived from the GAINS model, which provides fuel- and vehicle-type emission factors. CO_2 arises from fossil-fuel combustion and industrial conversion (e.g., unconventional oil upgrading), while non-CO_2 emissions from resource extraction are modeled nationally, agricultural and land-use emissions at the basin scale, and energy-related emissions at the state level.

State-level CH_4, N_2O, and F-gas trajectories follow the EPA's 2019 global non-CO_2 GHG Projections, and air-pollutant inventories (BC, OC, $PM_{2.5}$, PM_{10}, NOx, SO_2, NMVOCs, CO, and NH_3) are drawn from the EPA NEI and calibrated with global CEDS data. Annual state-level data from EPA Tier 1 Criteria Air Pollutants improve temporal continuity between NEI benchmark years, while national totals are scaled to CEDS for temporal consistency. Future emissions are released from the projected activity and technology-specific emission factors; non-CO_2 GHG abatement costs utilize EPA's 2019 marginal abatement cost curves, and pollutant factors account for sectoral regulations such as Tier 3 vehicle standards and New Source Performance Standards.

GCAM-USA applies existing standards: EPA's NSPS 2015 for electricity, Residential Wood Heater rules for buildings, MOVES 2014 Tier 3 and GHG vehicle regulations for on-road transport, GHGs, regulated emissions, and energy use in technologies (GREET 2014[26]) policy assumptions for industrial energy use, and GDP-based controls for industrial process, urban, and cement sectors.

S.1.H. Use of GCAM emission projections in COBRA

COBRA inputs include a version of the 2016 EPA National Emissions Inventory (NEI), as well as projections of that inventory to 2023 and 2028. EPA provides input files such that the impact of emissions changes can be evaluated in additional years as well, including 2030 through 2050 in 5-year increments. This inventory can be perturbed, with the embedded source-receptor matrix estimating air quality and impacts. The NEI is represented in COBRA at the county-level and aggregated by inventory "Tier", which groups similar sources.

The inventory in COBRA includes point sources, non-point stationary sources, and mobile sources. Point-sources encompass large, stationary emitters including major industrial installations, power plants, airports, and certain commercial or portable operations (e.g., asphalt plants, rock crushers). Some states also elect to report facilities like dry cleaners, gas stations, and livestock operations as nonpoint source emissions. In that case the numerous small emitters, such as residential heating systems, are aggregated at the county or tribal level because their individual emissions fall below reporting thresholds.

Mobile-source emissions are divided into on-road and non-road categories. On-road emissions derive from vehicles operating on public roadways, including light- and heavy-duty cars and trucks, idling, and refueling losses, and are estimated using EPA's MOVES model (or, in California, a state-specific model). These are split between the On-road and Nonpoint Data Categories in the Emissions Inventory System. Non-road mobile emissions cover off-road equipment powered by gasoline, diesel, or other fuels, such as construction machinery, lawn and garden tools, airport ground support vehicles, locomotives, and marine vessels. Since 2008, certain non-road sources (e.g., aircraft during takeoff and landing, locomotive operations at railyards) are reported as point sources, while others (underway marine and remaining locomotive emissions) are included as non-point sources. Wildland and agricultural fires, comprising prescribed burns, wildfires, and agricultural burns, are no longer treated as discrete events but are aggregated into annual, county-level totals within the non-point category, using satellite detections and activity data from state, local, tribal, and forestry agencies to inform the National Fire Emissions Inventory.

For each five-year interval, GCAM-USA produces state-level emissions of SO_2, NOx, $PM_{2.5}$, and NMVOC disaggregated by GCAM sectors and end-use technologies. We map aggregate emissions to COBRA's NEI-based source categories using the emissions mapping table provided in the supplementary data.

For each scenario, state, pollutant, and category, we divide future-year emissions by an interpolated 2023 value to calculate a set of emissions growth and control factors. Within a spreadsheet, these multiplicative factors are applied to adjust the COBRA 2023 inventory to a future year. COBRA operates by analyzing emissions changes in a particular year, comparing emissions between a control case and a reference case.

For this study, the reference case is NCA-USA and High-CDR and Low-CDR are evaluated individually as alternative cases. The years that were evaluated included 2025, 2030, 2035, 2040, 2045, and 2050.

PM- and O_3-related health outcomes included in our analysis are provided in Table S.5.

Table S.5. PM- and O3-related health outcomes included in this analysis

**$PM_{2.5}$-related Health Outcomes**	**O_3-related Health Outcomes**
Incidence, Asthma	
Incidence, Hay Fever/Rhinitis	
ER Visits, Respiratory	
Hospital Admits All Respiratory	
Mortality, All Cause	Mortality (Short term exposure)
Infant Mortality	Mortality (Long-term exposure)
Asthma Symptoms, Albuterol use	Asthma Symptoms, Chest Tightness
Nonfatal Heart Attacks	Asthma Symptoms, Cough
Minor Restricted Activity Days	Asthma Symptoms, Shortness of Breath
Work Loss Days	Asthma Symptoms, Wheeze
Incidence Lung Cancer	ER Visits, Asthma
Hospitalization Cardio Cerebro and Peripheral Vascular Disease	School Loss Days, All Cause
Hospitalization Alzheimers Disease	
Hospitalization Parkinsons Disease	
Incidence Stroke	
Incidence Out of Hospital Cardiac Arrest	
ER visits All Cardiac Outcomes	

S.2. Additional results

S.2.A. Global warming will be impacted by U.S. climate policy

Figure S.5 depicts projected global radiative forcing and global average temperature changes relative to the preindustrial levels (1850-1900) for the NCA-USA, Low CDR, and High CDR scenarios. The panel on the right integrates radiative forcing of the modelled decarbonization pathways into global mean surface temperature projections using the FaIR model and situates them against historical observations and four illustrative SSP reference scenarios (SSP1-2.6, SSP2-4.5, SSP3-7.0, and SSP5-8.5).

Results are calibrated to outputs of IPCC AR6 Working Group I–consistent probabilistic ensembles generated by FaIR v1.6.2, using parameter sets constrained by observed climate metrics in a large Monte Carlo ensemble. Historical temperature observations (black dashed line, grey shade, and projected dashed lines for the SSPs) are depicted using the datasets provided in synthesis report and summary for policymakers of the IPCC AR6 Working Group I.[27,28] We generated the historical purple line and the solid lines for the three modelled scenarios with FaIR using historical emissions for SSP2 and GCAM projected emissions. By 2050, NCA-USA exhibits the highest forcing across our scenarios, reaching approximately +3.59 Wm^{-2} radiative forcing and +1.74°C warming relative to the preindustrial levels. The High and Low CDR scenarios reduce the global radiative forcing by about 0.25 Wm^{-2} compared to the NCA-USA; therefore, the mid-century warming rises to about 1.65°C, amounting to 0.09°C reduction relative to NCA-USA.

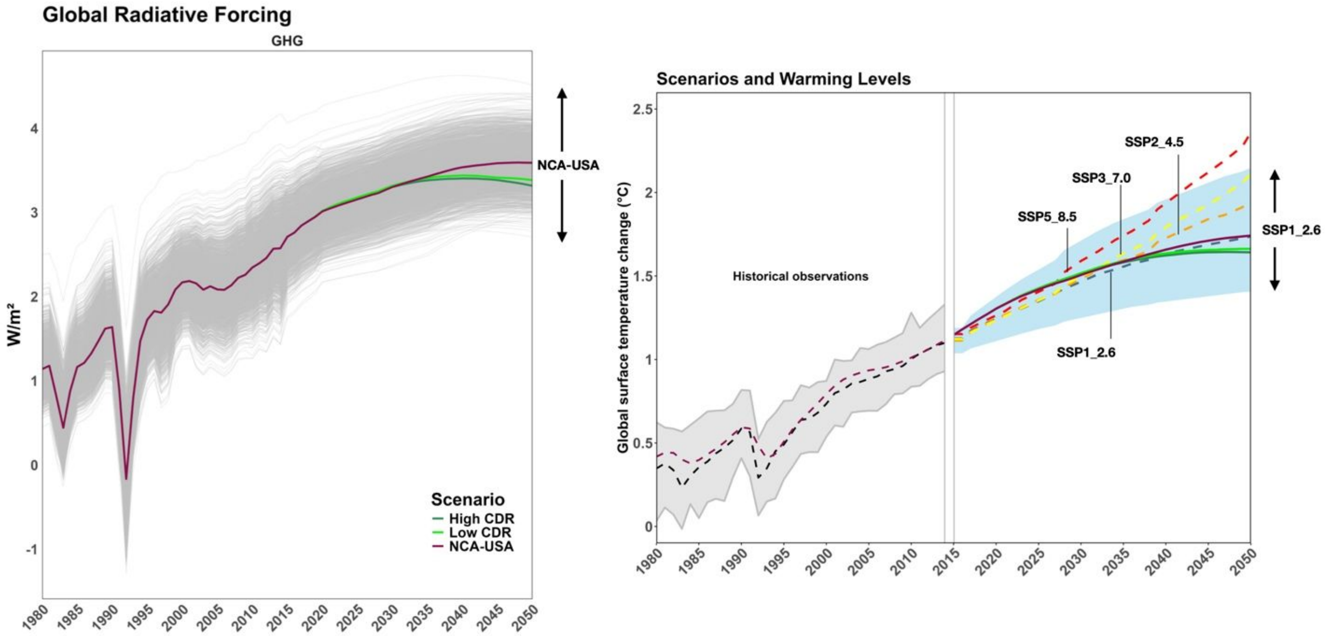

Figure S.5. Change in global radiative forcing and impact on global average temperature change relative to preindustrial (1850–1900) levels.

S.2.B. Emissions from electricity generation with CCS

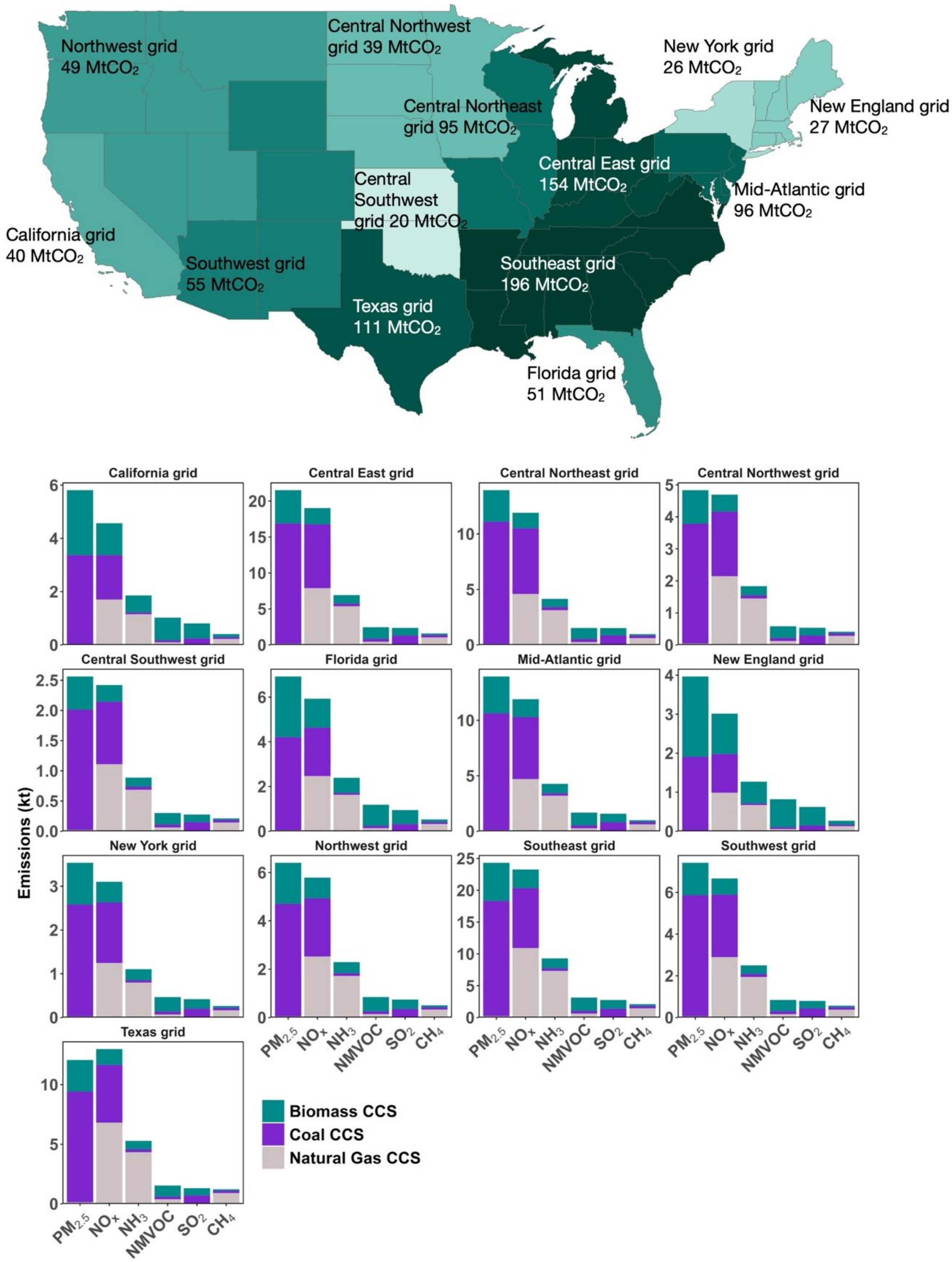

Figure S.6. CO_2 sequestration and associated emissions in electricity sector in 2050 - High CDR.

S.2.C. State-level criteria air pollution by scenario

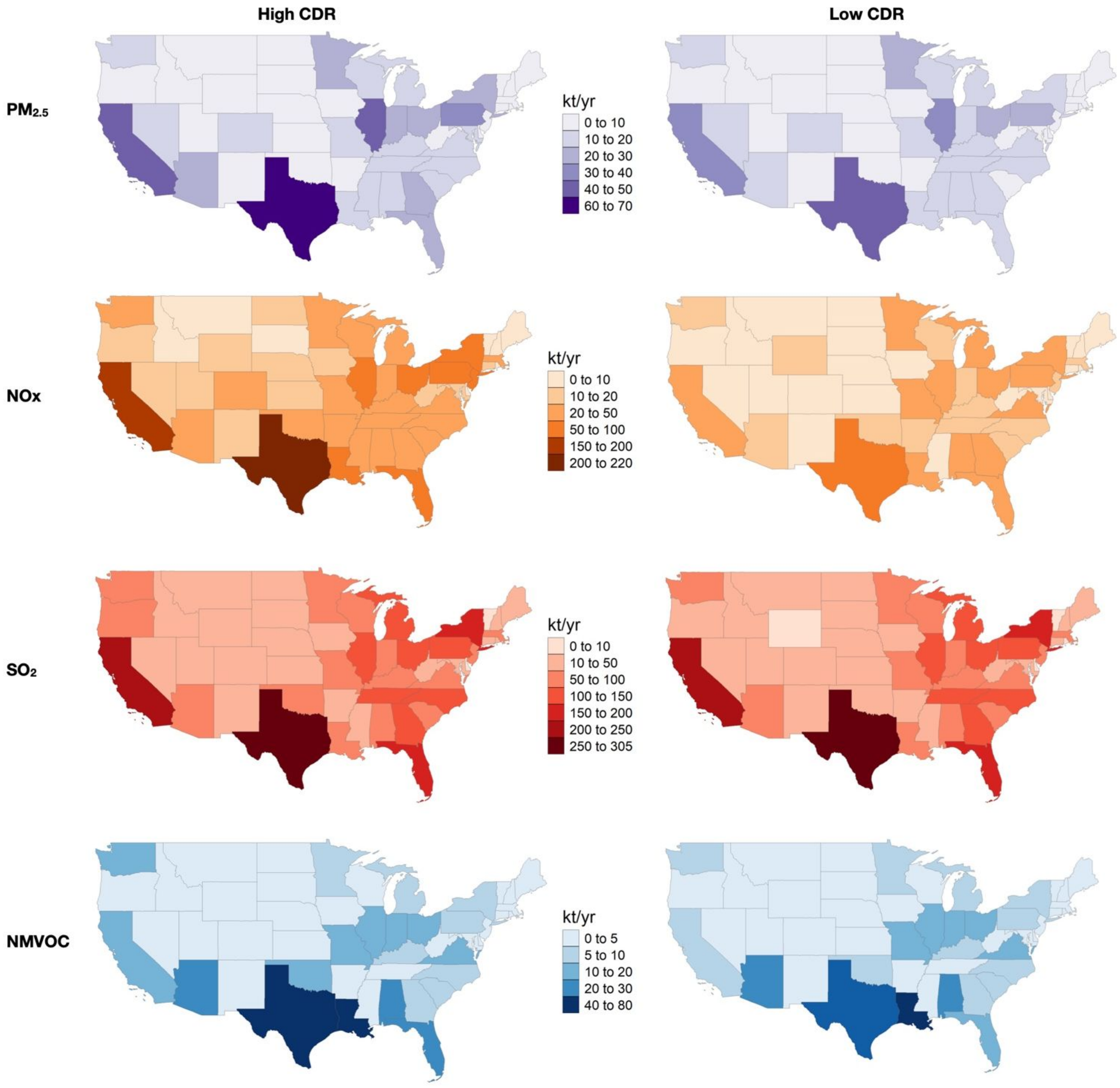

Figure S.7. State-level non-CO_2 emission in 2050.

S.2.D. Marginal abatement cost and deployment potential of CDR technologies in the U.S. under High-CDR scenario

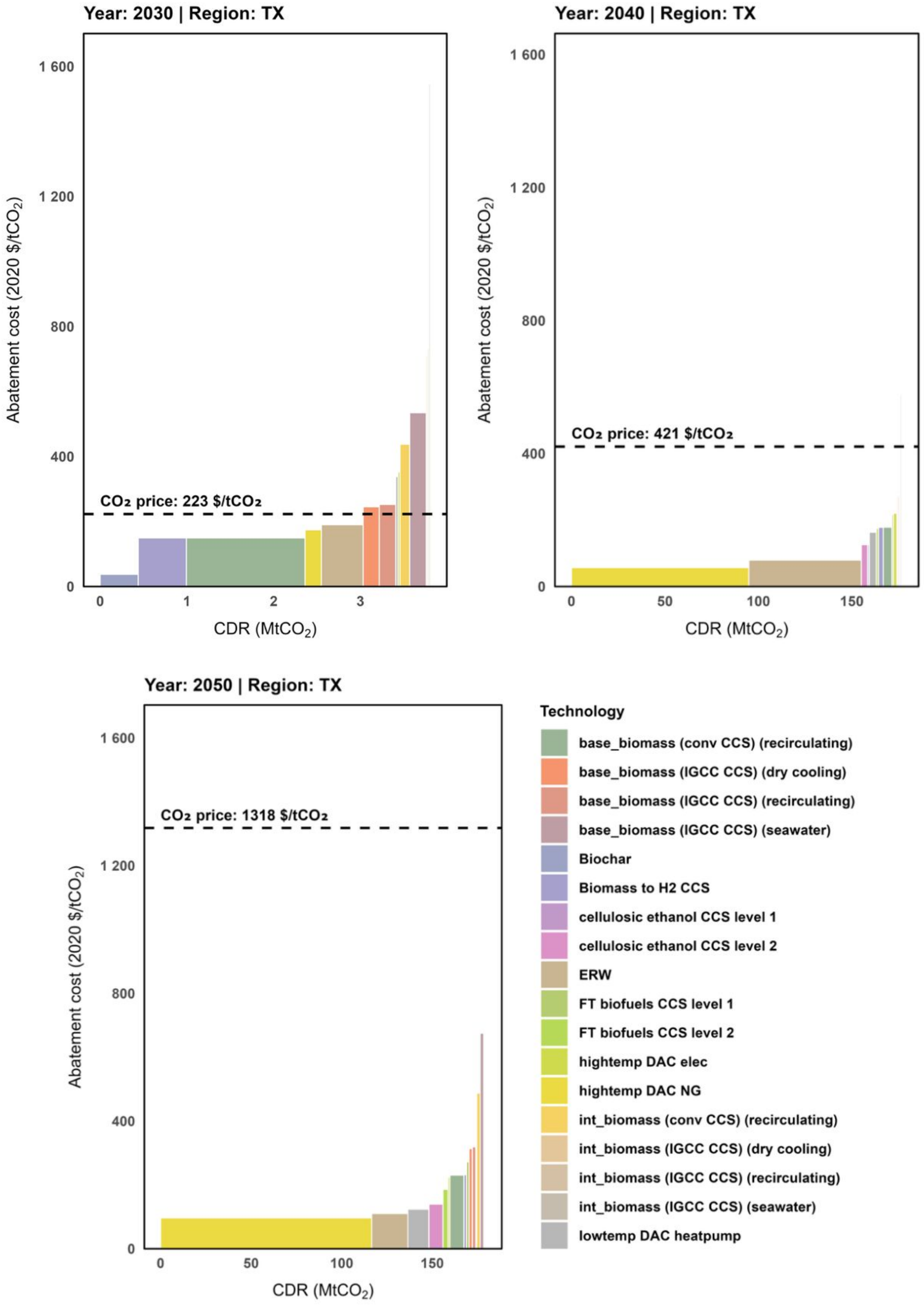

Figure S.8. Marginal abatement cost and deployment potential of CDR technologies in Texas under High-CDR scenario.

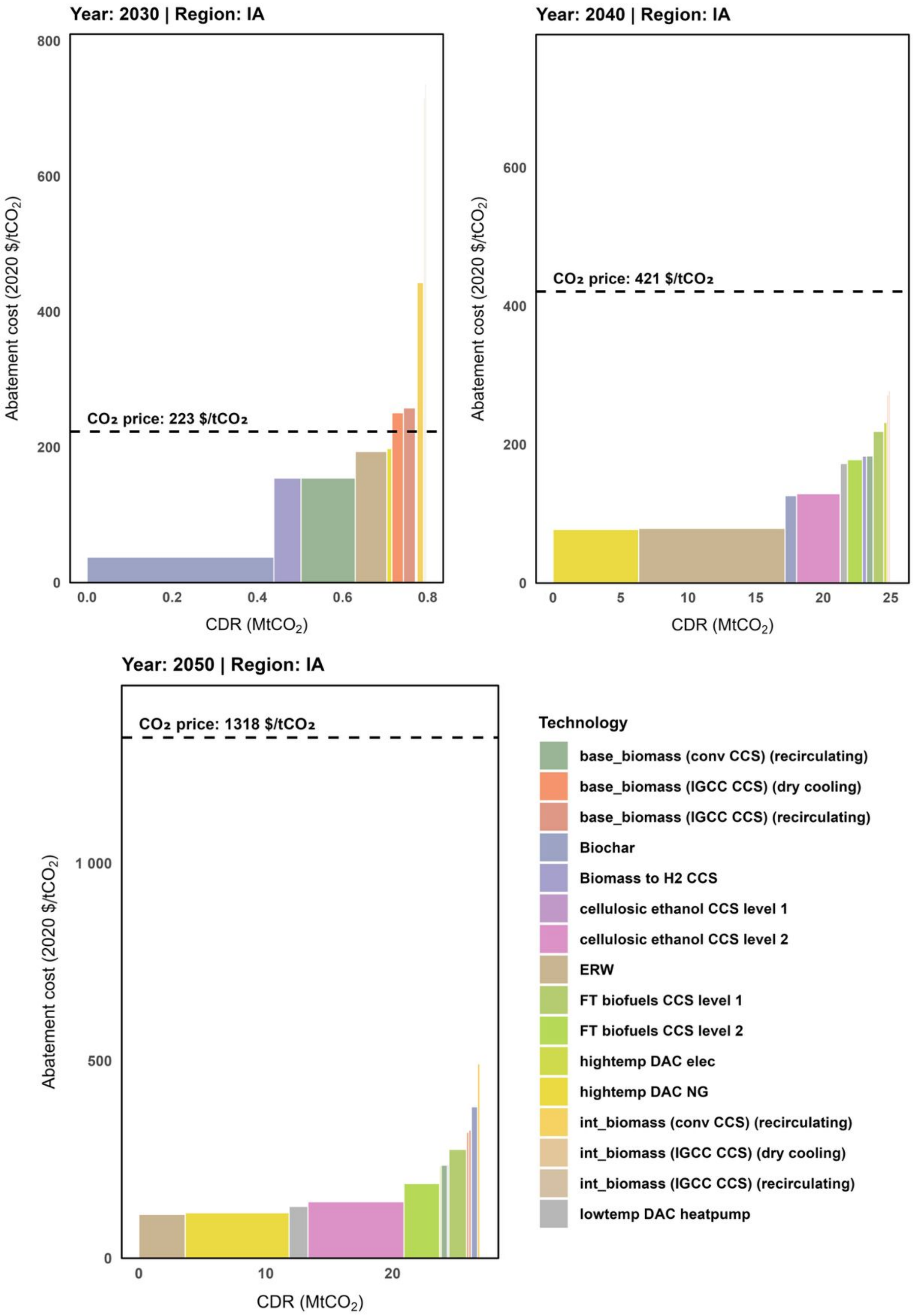

Figure S.9. Marginal abatement cost and deployment potential of CDR technologies in Iowa under High-CDR scenario.

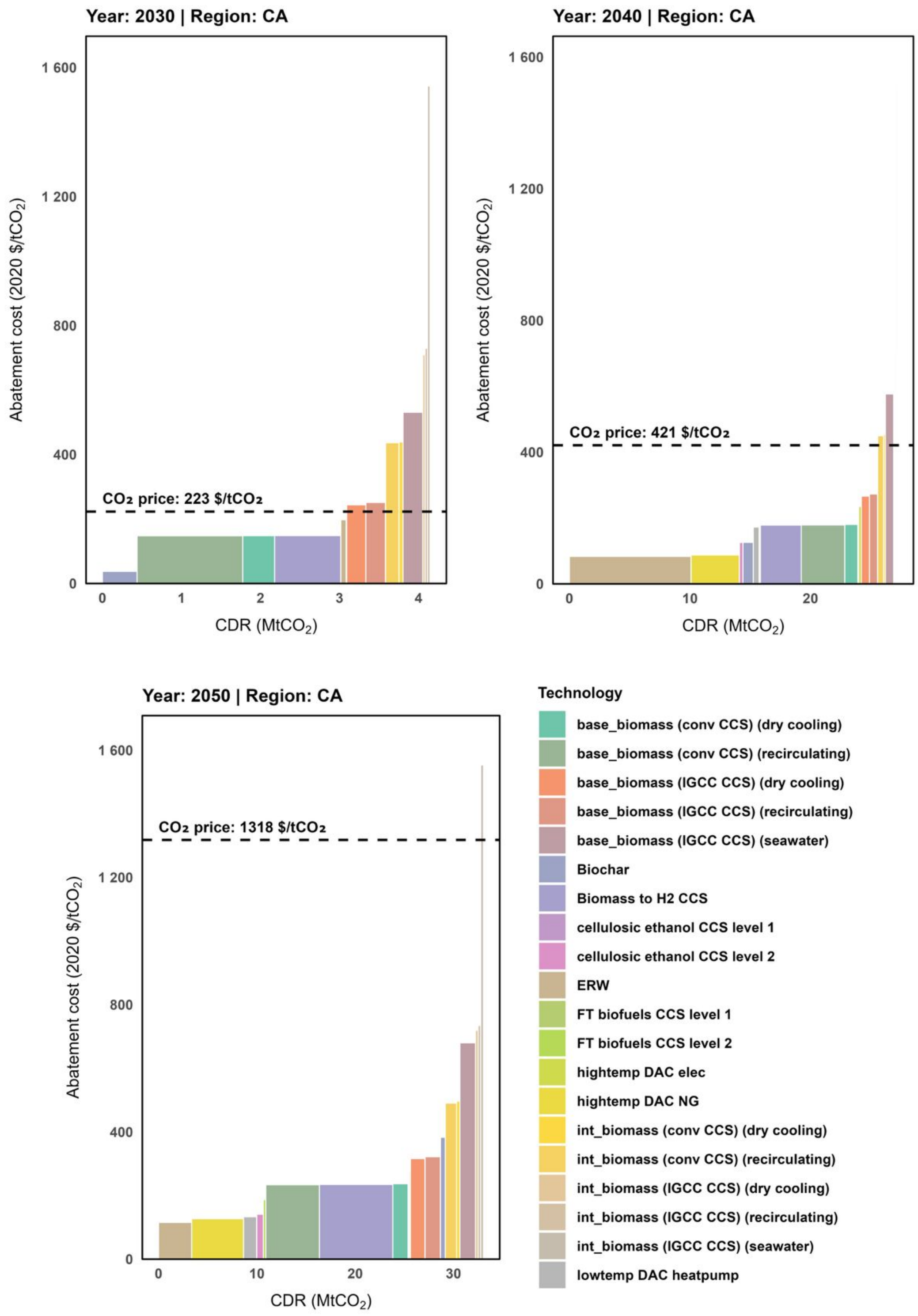

Figure S.10. Marginal abatement cost and deployment potential of CDR technologies in California under High-CDR scenario.

S.2.E. Cumulative discounted net CO_2 mitigation costs as a fraction of cumulative discounted GDP

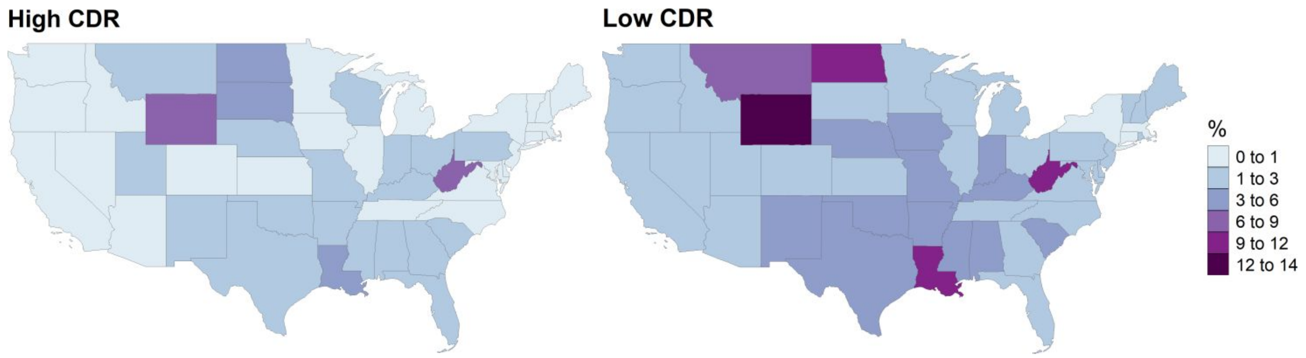

Figure S.11. Cumulative discounted net CO_2 mitigation costs as a fraction of cumulative discounted GDP (2025-2050) with an interest rate of 2%.

References

1. European Environment Agency. Air pollution impacts from carbon capture and storage (CCS). *EEA Technical report No 14/2011* (2011).
2. *EMEP/EEA Air Pollutant Emission Inventory Guidebook 2023 : Technical Guidance to Prepare National Emission Inventories*. (Publications Office of the European Union, 2023).
3. Khan, M. A. H. *et al.* The emissions of water vapour and NOx from modelled hydrogen-fuelled aircraft and the impact of NOx reduction on climate compared with kerosene-fuelled aircraft. *Atmosphere (Basel).* **13**, 1660 (2022).
4. U.S. EPA. AP 42, Fifth Edition, Volume I Chapter 13: Miscellaneous Sources. https://www.epa.gov/air-emissions-factors-and-quantification/ap-42-fifth-edition-volume-i-chapter-13-miscellaneous-0.
5. Global Wind Atlas. https://globalwindatlas.info/en/ (2025).
6. Bergero, C., Wise, M., Lamers, P., Wang, Y. & Weber, M. Biochar as a carbon dioxide removal strategy in integrated long-run mitigation scenarios. *Environmental Research Letters* **19**, 074076 (2024).
7. Roberts, K. G., Gloy, B. A., Joseph, S., Scott, N. R. & Lehmann, J. Life cycle assessment of biochar systems: estimating the energetic, economic, and climate change potential. *Environ. Sci. Technol.* **44**, 827–833 (2010).
8. Jeroen Kuenen, Ole-Kenneth Nielsen, Otto Rentz, Mike Woodfield & Robert Stewar. *1.A.2 Combustion in Manufacturing Industries and Construction 2023*. https://www.eea.europa.eu/publications/emep-eea-guidebook-2023/part-b-sectoral-guidance-chapters/1-energy/1-a-combustion/1-a-2-combustion-in/view (2023).
9. Strefler, J., Amann, T., Bauer, N., Kriegler, E. & Hartmann, J. Potential and costs of carbon dioxide removal by enhanced weathering of rocks. *Environmental Research Letters* **13**, (2018).
10. U.S. EPA. 11.19.2 Crushed Stone Processing and Pulverized Mineral Processing. https://www.epa.gov/sites/default/files/2020-10/documents/c11s1902.pdf (2004).
11. Fuhrman, J. *et al.* Diverse carbon dioxide removal approaches could reduce impacts on the energy–water–land system. *Nat. Clim. Chang.* **13**, 341–350 (2023).
12. Javadi, P. *et al.* The impact of regional resources and technology availability on carbon dioxide removal potential in the United States. *Environmental Research: Energy* **1**, 045007 (2024).
13. U.S. EPA. 5 Source Categories - Stone Quarrying, Crushing and Screening Facilities (Final Rule). https://www.epa.gov/tribal-air/5-source-categories-stone-quarrying-crushing-and-screening-facilities-final-rule (2024).
14. Dahowski, R. T., Dooley, J. J., Davidson, C. L., Bachu, S. & Gupta, N. Building the cost curves for CO2 storage: North America. *IEA Greenhouse Gas R&D Programme, Cheltenham, UK* (2005).
15. Dahowski, R. T., Davidson, C. L. & Dooley, J. J. Comparing large scale CCS deployment potential in the USA and China: A detailed analysis based on country-specific CO2 transport & storage cost curves. *Energy Procedia* **4**, 2732–2739 (2011).
16. Dahowski, R. T., Davidson, C. L., Li, X. C. & Wei, N. A $70/tCO2 greenhouse gas mitigation backstop for China's industrial and electric power sectors: Insights from a comprehensive CCS cost curve. *International Journal of Greenhouse Gas Control* **11**, 73–85 (2012).
17. Pett-Ridge, J. *et al.* Roads to Removal. *Options for Carbon Dioxide Removal in the United States* (2023).

18. Dai, T. *et al.* Land-based resources for engineered carbon dioxide removal in the United States exceed the expected needs. *One Earth* **8**, (2025).
19. Bradley, R. A., Watts, E. C. & Williams, E. R. *Limiting Net Greenhouse Gas Emissions in the United States*. (1991).
20. Joint Global Change Research Institute, P. N. N. L. GCAM v7.1 Documentation: GCAM Policies. https://jgcri.github.io/gcam-doc/policies.html (2025).
21. Fricko, O. *et al.* The marker quantification of the Shared Socioeconomic Pathway 2: A middle-of-the-road scenario for the 21st century. *Global Environmental Change* **42**, 251–267 (2017).
22. Vimmerstedt, L. *et al. 2019 Annual Technology Baseline (ATB) Cost and Performance Data for Electricity Generation Technologies*. (2019).
23. Mai, T. T. *et al. Electrification Futures Study: Scenarios of Electric Technology Adoption and Power Consumption for the United States*. (2018).
24. Hoesly, R. M. *et al.* Historical (1750–2014) anthropogenic emissions of reactive gases and aerosols from the Community Emissions Data System (CEDS). *Geosci. Model Dev.* **11**, 369–408 (2018).
25. Global Fire Emissions Database (GFED). https://www.globalfiredata.org/.
26. Argonne National Laboratory. 2014. GREET v1.2.0 11425. https://greet.anl.gov/.
27. Fyfe, J., Fox-Kemper, B., Kopp, R. & Garner, G. Summary for policymakers of the working group I contribution to the IPCC sixth assessment report-Data for figure SPM. 8 (v20210809). *(No Title)* (2021).
28. Lee, H. *et al. Climate Change 2023: Synthesis Report. Contribution of Working Groups I, II and III to the Sixth Assessment Report of the Intergovernmental Panel on Climate Change*. (The Australian National University, 2023).
